# Crystal Structure of Cytomegalovirus IE1 Protein Reveals Targeting of TRIM Family Member PML via Coiled-Coil Interactions

**DOI:** 10.1371/journal.ppat.1004512

**Published:** 2014-11-20

**Authors:** Myriam Scherer, Stefan Klingl, Madhumati Sevvana, Victoria Otto, Eva-Maria Schilling, Joachim D. Stump, Regina Müller, Nina Reuter, Heinrich Sticht, Yves A. Muller, Thomas Stamminger

**Affiliations:** 1 Institute for Clinical and Molecular Virology, University of Erlangen-Nuremberg, Erlangen, Germany; 2 Division of Biotechnology, University of Erlangen-Nuremberg, Erlangen, Germany; 3 Division of Bioinformatics, Institute of Biochemistry, University of Erlangen-Nuremberg, Erlangen, Germany; University of Glasgow, United Kingdom

## Abstract

PML nuclear bodies (PML-NBs) are enigmatic structures of the cell nucleus that act as key mediators of intrinsic immunity against viral pathogens. PML itself is a member of the E3-ligase TRIM family of proteins that regulates a variety of innate immune signaling pathways. Consequently, viruses have evolved effector proteins to modify PML-NBs; however, little is known concerning structure-function relationships of viral antagonists. The herpesvirus human cytomegalovirus (HCMV) expresses the abundant immediate-early protein IE1 that colocalizes with PML-NBs and induces their dispersal, which correlates with the antagonization of NB-mediated intrinsic immunity. Here, we delineate the molecular basis for this antagonization by presenting the first crystal structure for the evolutionary conserved primate cytomegalovirus IE1 proteins. We show that IE1 consists of a globular core (IE1_CORE_) flanked by intrinsically disordered regions. The 2.3 Å crystal structure of IE1_CORE_ displays an all α-helical, femur-shaped fold, which lacks overall fold similarity with known protein structures, but shares secondary structure features recently observed in the coiled-coil domain of TRIM proteins. Yeast two-hybrid and coimmunoprecipitation experiments demonstrate that IE1_CORE_ binds efficiently to the TRIM family member PML, and is able to induce PML deSUMOylation. Intriguingly, this results in the release of NB-associated proteins into the nucleoplasm, but not of PML itself. Importantly, we show that PML deSUMOylation by IE1_CORE_ is sufficient to antagonize PML-NB-instituted intrinsic immunity. Moreover, co-immunoprecipitation experiments demonstrate that IE1_CORE_ binds via the coiled-coil domain to PML and also interacts with TRIM5α We propose that IE1_CORE_ sequesters PML and possibly other TRIM family members via structural mimicry using an extended binding surface formed by the coiled-coil region. This mode of interaction might render the antagonizing activity less susceptible to mutational escape.

## Author Summary

Research of the last few years has revealed that microbial infections are not only controlled by innate and adaptive immune mechanisms, but also by cellular restriction factors, which give cells the capacity to resist pathogens. PML nuclear bodies (PML-NBs) are dot-like nuclear structures representing multiprotein complexes that consist of the PML protein, a member of the TRIM family of proteins, as well as a multitude of additional regulatory factors. PML-NB components act as a barrier against many viral infections; however, viral antagonistic proteins have evolved to modify PML-NBs, thus abrogating this cellular defense. Here, we delineate the molecular basis for antagonization by the immediate-early protein IE1 of the herpesvirus human cytomegalovirus. We present the first crystal structure for the evolutionary conserved core domain (IE1_CORE_) of primate cytomegalovirus IE1, which exhibits a novel, unusual fold. IE1_CORE_ modifies PML-NBs by releasing other PML-NB proteins into the nucleoplasm which is sufficient to antagonize intrinsic immunity. Importantly, IE1_CORE_ shares secondary structure features with the coiled-coil domain (CC) of TRIM factors, and we demonstrate strong binding of IE1 to the PML-CC. We propose that IE1_CORE_ sequesters PML and possibly other TRIM family members via an extended binding surface formed by the coiled-coil domain.

## Introduction

Promyelocytic leukemia protein PML is the organizer of small nuclear matrix structures termed nuclear bodies (NBs) or nuclear domain 10 (ND10) [1]. PML, also named TRIM19, is a member of the tripartite motif (TRIM) family of proteins, which are characterized by the presence of RING, B-box and coiled-coil domains [2]. Recent studies showed that an unprecedented large number of TRIMs positively regulate innate immune signaling pathways by acting as E3-Ub ligases [3], [4]. Additionally, a subgroup of TRIMs, including PML, was demonstrated to exhibit small ubiquitin related modifier (SUMO) E3 activity and PML itself is covalently conjugated to SUMO on three lysine residues [5], [6]. This modification, which affects PML localization, stability and interaction with other partners, is critical for NB functions [7]. In response to stimuli, PML-NBs recruit a number of proteins implicated in different cellular processes such as DNA damage response, apoptosis, senescence and protein degradation [8], [9].

Accumulating evidence implicates this subnuclear structure as an important component of intrinsic immunity against viruses from different families including herpes-, adeno-, polyoma, rhabdo- and retroviruses [10]–[12]. Unlike the innate and adaptive immunity, the intrinsic immune response is mediated by cellular restriction factors that are constitutively expressed and permanently active, even before a pathogen enters the cell. Other characteristics of intrinsic immune mechanisms are that they are saturable and subject to viral countermeasures [13]. Besides PML, a number of NB components, such as Sp100, hDaxx and ATRX, function as cellular restriction factors. Recent evidence suggests that NB proteins independently contribute to the repression of herpesvirus replication, raising the concept that individual NB components, rather than the PML-NB structure as a whole, restrict viral infections [14]–[20]. Consequently, various viruses have been shown to antagonize the intrinsic cellular defense via the modification of NB proteins. For instance, the herpes simplex virus type I immediate-early protein ICP0 has been described as a viral ubiquitin ligase with preferential substrate specificity for SUMO-modified isoforms of PML thus promoting the degradation of PML [21]. However, no structural information on this and many other NB-antagonistic proteins is available, yet.

Human cytomegalovirus (HCMV), a ubiquitous beta-herpesvirus causing serious disease in immunocompromised individuals, encodes an abundant immediate-early protein termed IE1 that modulates innate immune mechanisms as well as other cellular processes (reviewed in [22]). Although IE1 is a major player in the initiation of lytic HCMV infection and has been subject to extensive studies over the last decades, structural data on this protein are still limited. Four distinct regions have been identified within the 491 amino acid IE1 protein: a short N-terminal region that is required for nuclear import, a large core domain, an acidic region near the C-terminus that harbors a SUMOylation site and a 16 amino acid chromatin-tethering domain (CTD) at the extreme C-terminus [23]–[26]. Recent results have suggested that the acidic C-terminal region of IE1 is characterized by a lack of well-defined three-dimensional structure, but contains a binding motif for signal transducer and activator of transcription (STAT) proteins. This interaction site enables IE1 to compromise STAT-mediated interferon signaling, thereby interfering with a crucial branch of the innate immune system and promoting viral replication [27]–[29]. In addition to its effects on the innate immune system, IE1 is required to overcome the PML-NB-mediated intrinsic immunity that targets HCMV immediately upon infection. IE1 transiently co-localizes with PML-NBs during the first 2–4 hours after infection but subsequently induces disruption of these structures [30]–[32]. NB dispersal correlates with the functional activities of IE1 during infection and a PML knock-down efficiently compensates for IE1 in promoting replication of an IE1-deficient virus, establishing IE1 as an important antagonist of PML-mediated cellular repression of viral replication [15], [16], [33]. Studies on the mechanism of NB dispersal have demonstrated that IE1 induces the loss of the SUMOylated forms of PML, and also influences the SUMOylation state of Sp100 [34], [35]. However, in contrast to ICP0, this neither requires proteasomal activity nor does IE1 affect the abundance of unmodified PML [24], [35]. In further studies, a physical interaction between IE1 and PML, which requires the N-terminal TRIM region of PML, has been detected as prerequisite for the transient co-localization and subsequent disruption of PML-NB integrity. The interaction site for PML has been mapped to the large core region of IE1, since deletions or mutations affecting this domain abrogate PML binding and NB disruption [23], [35], [36]. However, it was noted in several reports that mutations in the core region often result in unstable IE1 proteins, so that the molecular basis for the IE1-PML interaction remains uncharacterized [37], [38].

Here we report the crystal structure of the evolutionary conserved globular core domain of primate cytomegalovirus IE1 proteins, determined to 2.3 Å resolution. Unexpectedly, the overall structure does not resemble any known protein fold, but exhibits an unusual all α-helical, femur-like shape which shares secondary structure features recently observed in the coiled-coil domain of TRIM proteins. We show that this IE1_CORE_ domain binds with high affinity to PML via the coiled-coil domain. This induces PML de-SUMOylation thus releasing the PML-associated factors hDaxx, Sp100 and ATRX, while PML accumulations itself are not dispersed. Since IE1_CORE_ efficiently complements lytic replication of an IE1-deleted HCMV, we conclude that sequestration of PML via IE1_CORE_ is sufficient for antagonization of NB-mediated intrinsic immunity. Thus, cytomegaloviruses may have evolved a distinct structural fold to effectively bind and neutralize an important cellular hub protein that exerts critical roles during the regulation of innate immune responses as well as the control of programmed cell death [8]–[10], [12].

## Results

### IE1 consists of a globular core domain flanked by intrinsically disordered regions

**Figure 1:**
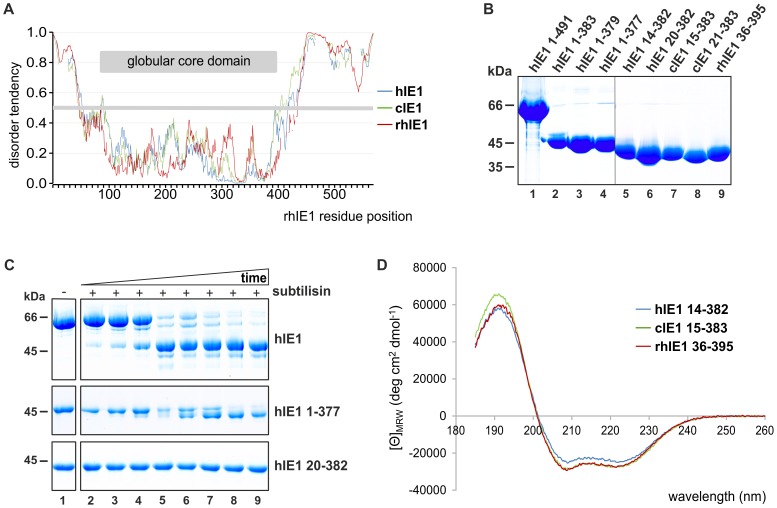
The IE1 proteins of the primate cytomegaloviruses human (h), chimpanzee (c) and rhesus (rh) cytomegalovirus contain a conserved, stably folded globular core domain. (A) *In silico* analysis of the hIE1, cIE1 and rhIE1 sequences using IUPred [39]. Disorder score for IE1 suggesting the presence of a central folded globular domain (scores <0.5; gray box). Scores ≥0.5 (gray line) indicate disorder. (B) Prokaryotic expression and purification of hIE1, cIE1 and rhIE1 proteins used for crystallization attempts. The panel shows a Coomassie blue stained SDS-PAGE of the purified proteins as indicated. (C) Experimental confirmation of the folded globular IE1 domain (IE1_CORE_) by limited proteolysis. Full-length (1–491), C-terminally truncated (1–377) or N/C-terminally truncated (20–382) hIE1 protein was incubated with subtilisin and analyzed at different time points (e.g. at 1, 10, 30, 60, 120, 180, 240 and 300 min) by SDS-PAGE and Coomassie blue staining. (D) Circular dichroism analysis of the hIE1(14–382), cIE1(15–383) and rhIE1(36–395) core domains indicating a conserved, highly α-helical fold.

In order to further clarify the mechanism of IE1-mediated PML antagonization, we investigated the molecular architecture of the IE1 proteins from human, chimpanzee and rhesus cytomegalovirus (h-, c- and rhIE1) (Figure S1). As previously proposed by Krauss *et al.* [28], *in silico* predictions using the web server IUPred [39] suggested that the N- and C-terminal regions of all IE1 proteins display consistently high intrinsic disorder propensities (Figure 1A). Based on these predictions as well as on sequence conservation and the characterization of protease-resistant IE1 subdomains, we generated truncated IE1 constructs covering the folded core (Figure 1B). Limited proteolysis of recombinant full-length hIE1 as well as of C- or N/C-terminally truncated hIE1 proteins confirmed the *in silico* predictions (Figure 1C). These studies revealed the existence of a stably folded IE1_CORE_ domain of about 360 residues (Figure 1C, hIE1 20-382) that is flanked at the N- and C-termini by intrinsically disordered regions (IE1_N-IDR_ and IE1_C-IDR_). Circular dichroism (CD) spectroscopy [40], [41] was applied to investigate the secondary structure composition of the IE1 variants. All hIE1 proteins produced typical α-helical spectra with negative ellipticity above 200 nm and two distinct minima at 208 nm and 222 nm (Figure 1D and S2). The spectra of full-length hIE1 and hIE1_CORE_ differed in the region below 210 nm. Calculation of the difference spectrum revealed strong random coil characteristics thereby confirming the predicted predominantly disordered nature of the terminal regions (Figure S2).

Sequence identities between 24 and 73% between h-, c- and rhIE1_CORE_ domains suggest that the core domains share identical folds. Indeed, the CD spectra of the recombinant proteins hIE1_CORE_, cIE1_CORE_ and rhIE1_CORE_ match extremely well and indicate that all core domains consist mainly of α-helical segments (Figure 1D).

### Crystal structure and architecture of IE1

**Figure 2:**
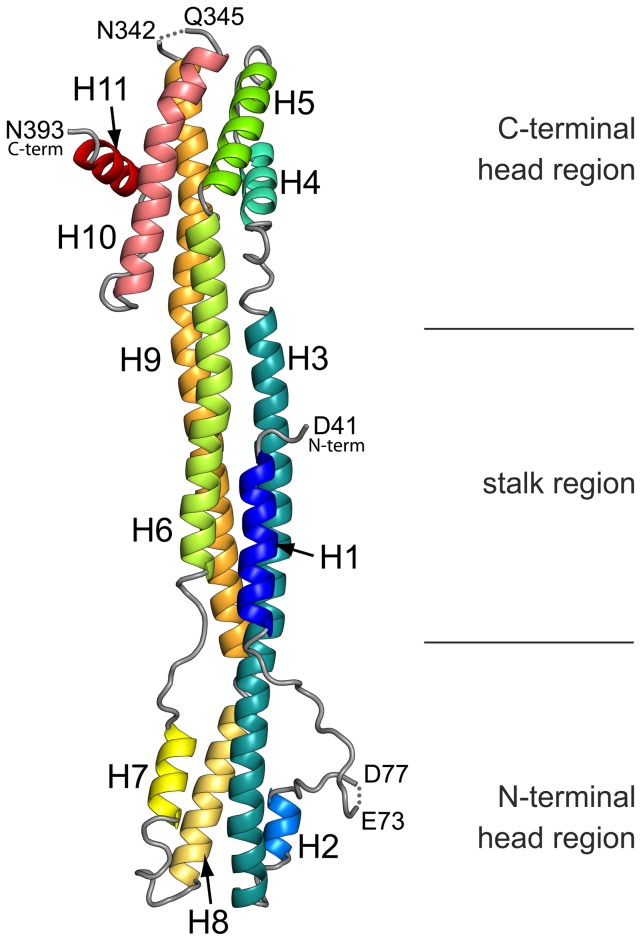
Structure of IE1_CORE_. Ribbon representation of the rhIE1 monomer (residues 41–393) illustrating the elongated, femur-like fold of IE1. The eleven α-helices (H1 to H11) are colored from blue to red. The location of the two head regions and the central stalk are indicated by black lines.

Crystallization trials with full-length hIE1 remained unsuccessful and, in case of the IE1_CORE_ variants, yielded suitable crystals only for rhIE1_CORE_ after chemical methylation of surface exposed lysine residues. The structure of rhIE1_CORE_ was solved using experimental phases and refined to 2.3 Å resolution (R_work_ = 19.73%, R_free_ = 24.96%) (Figures 2 and S3; Methods and Table S1). The main chain of the model was traced between amino acids 41 and 393. Since no well-defined electron density was visible for residues preceding residue 41 or following residue 393, we conclude that the core domain spans at least amino acids 42 to 392 of rhIE1 corresponding to residues 27 to 379 of hIE1. RhIE1_CORE_ consists of a total of 11 α-helices (Figure 2 and S1). Helices H3 and H9 are unusually long and contain as many as 16 and 17 helical turns, respectively. RhIE1_CORE_ adopts an elongated, femur-like shape with dimensions of 130×25×25 Å^3^.

The structure can be divided into three distinct regions, namely an N-terminal head region (rhIE1: residues 62–118, 236–283; hIE1 residues 46–103, 221–267) and a C-terminal head region (rhIE1 residues 151–207, 315–393; hIE1 residues 136–192, 300–380) interconnected by a stalk region (rhIE1 residues 41–61, 119–150, 208–235, 284–314; hIE1 residues 27–45, 104–135, 193–220, 268–299) (Figure 2). The stalk consists of an uncommon right-handed three-helix coiled-coil (α-helices H3, H6 and H9) with the N-terminal helix H1 added to one side of the three-helix bundle (Figure 2). The right-handed pairing of the helices goes in hand with the presence of hendecad repeats in the sequences of these helices [42]. In these repeats of 11 residues (numbered alphabetically *abcdefghijk*) the hydrophobic amino acids at positions *a*, *d*, and *h* are interspaced by 2 (*bc*), 3(*efg*) and 3(*ijk*) amino acids of predominantly polar nature (Figure S1). In contrast, the patterning of hydrophobic residues within the head regions of rhIE1_CORE_ frequently resembles that observed in heptad repeats (residue labeling *abcdefg*). Here the hydrophobic amino acids at positions *a* and *d* are interspaced by 2 (*bc*) and 3(*efg*) polar residues, and the interdigitation of the *a* and *d* residues from neighboring helices gives rise to more commonly observed left handed coiled-coil supersecondary structure elements [42]. Hence, both head regions display left-handed coiled-coils. Whereas the N-terminal head region consists of a three-helix bundle (α-helices H3, H7 and H8) with an additional helix H2 added onto one side of this bundle, the C-terminal head-region comprises 5-helical segments (H4, H5, H9, H10 and H11) in total. These can be grouped into two pairs of antiparallel left-handed coiled coils (H4–H5 and H9–H10) and an additional C-terminal helix (H11). The coiled-coil helix pairs pack against each other with crossing angles of approximately 50°, and thus the interactions between these coiled-coils resemble the ridges into grooves side chain packing observed in globins [43].

RhIE1_CORE_ also displays an extended loop region between helices H1 and H2 (residues 62 to 82) devoid of secondary structure elements. The conformation of this loop region is stabilized through extensive crystal packing contacts and differs between the two monomers of the dimeric unit as observed within the crystal (see below). Overall, the structure of rhIE1_CORE_ is in full agreement with the observed CD spectrum. Since, with the exception of the very terminal helices (H1, H2, H10 and H11), all intervening helical segments span the entire length of the molecule, IE1_CORE_ is described best as consisting of a single contiguous domain (Figure 2).

### IE1 displays a distinct fold that exhibits local similarity to the coiled-coil region of TRIM proteins

A search for structurally similar proteins revealed only partial hits that cover less than 50% of the total rhIE1_CORE_ length. The top-scoring hits belong to a considerable variety of domain folds which either contain α-helical orthogonal bundles or up-down bundles that partly resemble the IE1 head or stalk region, respectively (Table S2). This indicates that IE1 cannot readily be assigned to any known topology and suggests that the overall fold of IE1 is so far unique.

**Figure 3:**
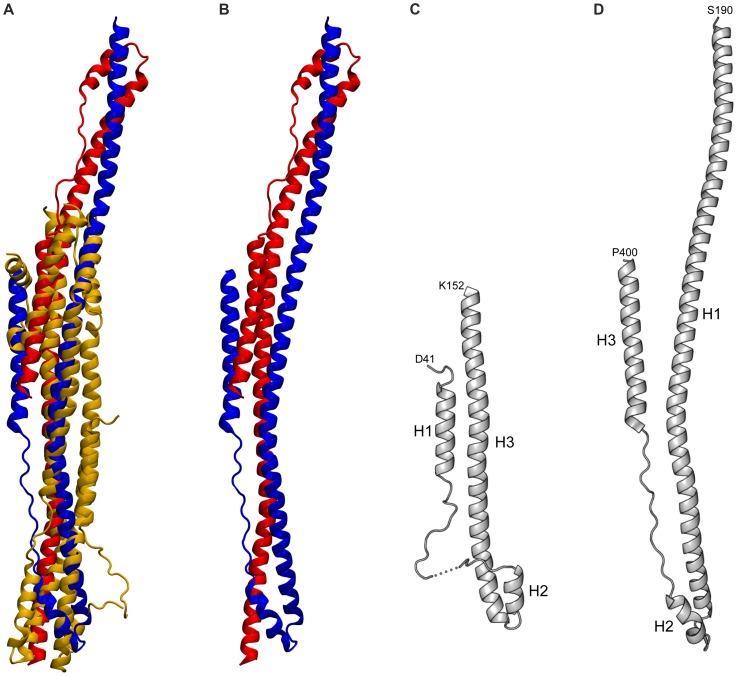
Structural comparison of the rhIE1 with TRIM25. (A) Overlay of dimeric TRIM25 (in blue and red) with monomeric rhIE1 (gold). (B) Dimeric TRIM25. (C) Topological arrangement of helices H1 to H3 of rhIE1. (D) Structure of the TRIM25 monomer.

An extended search for local structural similarities, which also considered multimeric proteins, revealed a similarity between IE1 and the recently described coiled-coil region of homodimeric TRIM25 (Figure 3) [44]. The coiled-coil region of TRIM25 is composed of three helices in which the long helices H1 and H1' (from the second monomer) align in an antiparallel fashion to form the TRIM25 dimer (Figure 3B). The H1/H1' helix pair can be superimposed onto rhIE1_CORE_ such that the helices superimpose with the H5/H6 and H8/H9 helices of IE1. The crystal structure of TRIM25 is also highly similar to that of two further TRIM family members, namely TRIM69 [45] and TRIM5α [46]. These structures suggest that the coiled-coil topology may be conserved among the entire family of TRIM proteins, possibly extending to PML (TRIM19), the target protein of IE1. This is further corroborated by sequence comparisons, demonstrating that TRIM family members, including PML, exhibit a distinct pattern of heptad and hendecad repeats for helix H1 (Figure S4) [44].

Interestingly, the topological arrangement of helices H1 to H3 of rhIE1 closely resemble the topology of helices H1 to H3 in TRIM25 when allowing for an inversion of the sequential order of the helices (Figures 3C and D). This also extends to the joint presence of heptad and hendecad repeats in H1 in TRIM25 and H3 in rhIE1. Whereas in TRIM25, the hendecad repeats occur in the central segment of helix H1 and are flanked on both sides by heptad repeats, H3 in rhIE1 displays a number of heptad repeats towards its N-terminus and switches to a segment of hendecad repeats that covers the second half of helix H3 (Figures S1 and S4). Taken together, sequence comparisons and three available coiled-coil structures demonstrate that the pattern of heptad and hendecad repeats is highly conserved across the TRIM protein family, and is also present in the viral IE1 protein. These observations suggest a common architecture of TRIM coiled-coils and provide evidence for structural similarities between IE1 and TRIM proteins.

### Oligomerization and conservation of primate cytomegalovirus IE1 proteins

**Figure 4:**
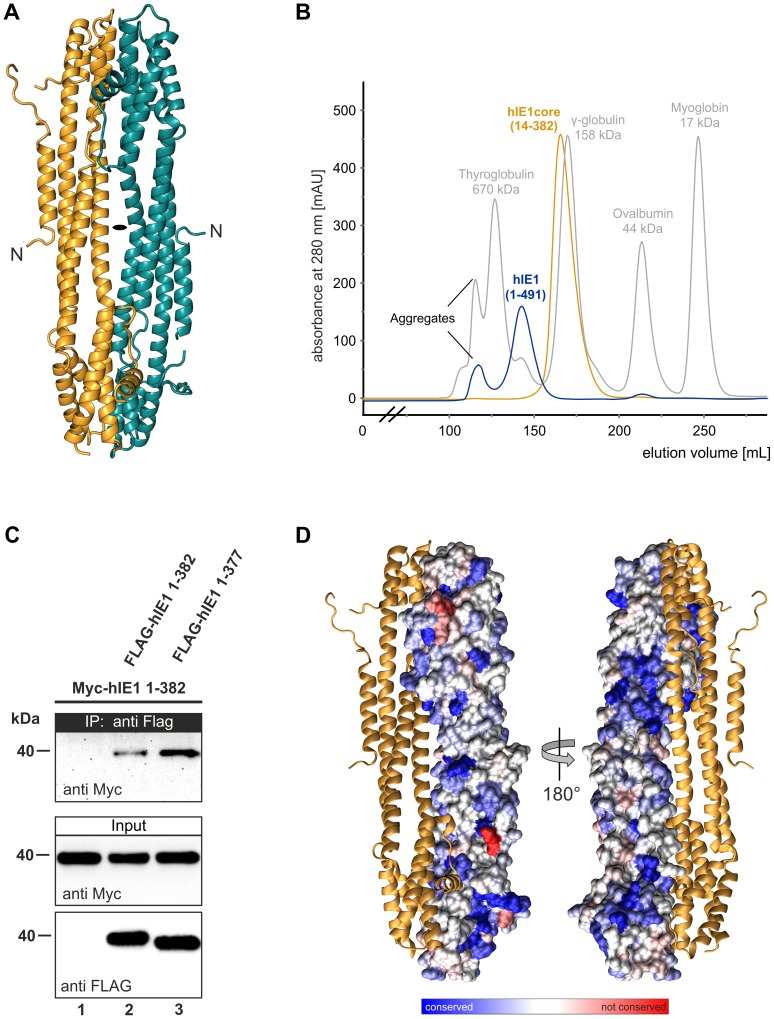
Oligomerization of IE1. (A) Dimeric assembly of rhIE1 in the crystal. The two monomers (in cyan and gold) are related by a local 2-fold rotation axis indicated by a black ellipse. (B) Self-association of recombinant IE1 and IE1_CORE_ in gel filtration experiments. Overlay of the elution profiles of full length hIE1(1–491) (blue) and hIE1_CORE_(14–382) (gold). Full length hIE1 and hIE1_CORE_ eluted as symmetric main peaks with apparent molecular weights of ∼390 kDa and ∼194 kDa, respectively. It currently remains unclear whether the elution behavior of hIE1_CORE_ merely reflects the elongated molecular shape of the dimer as observed in the crystal structure of rhIE1_CORE_ or whether hIE1_CORE_ assembles in solution into oligomers that comprise more than two molecules. Molecular weight estimates are based on commercially available calibration proteins, as indicated by the grey curve. (C) Self-interaction of the IE1_CORE_ in human cells. HEK293T cells were co-transfected with expression plasmids encoding Myc-tagged hIE1(1–382) and FLAG-tagged hIE1 variants comprising residues 1–382 or 1–377. After cell lysis, immunoprecipitation was performed with an anti-FLAG antibody. Proteins within the cell lysate (input) and co-precipitated PML (IP) were analyzed by Western blotting as indicated. (D) Evolutionary sequence conservation of IE1 mapped onto the surface of rhIE1. The chains of dimeric rhIE1 are shown in space-filled and ribbon representation, respectively. Residues are colored according to their degree of conservation ranging from highly conserved (dark blue) to highly variable (red). The two views differ by a rotation of 180° around a vertical axis.

RhIE1_CORE_ forms a dimer with C2 point group symmetry in the crystal (Figure 4A), and oligomerization of rhIE1 and hIE1 was confirmed both by gel filtration experiments and co-immunoprecipitation analyses (hIE1) (Figure 4B and C). RhIE1_CORE_ dimerizes with both stalk regions juxtaposed in an antiparallel fashion (Figure 4A). The main-chain conformation differs in the two monomers (Cα-RMSD for all helical segments  = 2.13 Å, Figure S5). This deviation originates from a pronounced kink that is observed in one of the two monomers and that causes a repositioning of the loop that interconnects helices H8 to H9 with a concomitant displacement of the N-terminal half of helix H9 (Figure S6). This kink solely occurs in the tetragonal crystal form whereas in the monoclinic space group all four monomers in the asymmetric unit are highly similar. Since this space group transition is triggered by the experimental dehydration of the crystals, we propose that this *in situ* molecular shaping reflects an inherent flexibility of the IE1_CORE_ fold that allows for small readjustments in the packing of the helices.

Intermolecular contacts are formed along the entire rhIE1 length resulting in an extraordinary large interface area (Ø 3173 Å^2^ per molecule). In the stalk region, these contacts are predominantly hydrophilic, whereas several sparse hydrophobic patches are formed between head regions. Because of its predominantly hydrophilic nature, the dimer interface does not resemble interfaces typically observed in permanent oligomers, suggesting that the dimer could become disrupted upon interaction with binding partners.

This idea is further corroborated by the analysis of IE1_CORE_ surface conservation indicating that the dimer interface is not higher conserved than the solvent exposed regions (Figure 4D). Evolutionary conserved surface patches are distributed almost over the entire surface of IE1_CORE_ (Figure 4D, blue), whereas non-conserved patches are mainly restricted to loop regions (Figure 4D, red). This indicates that the overall biophysical properties are conserved within the IE1 family of proteins despite the rather low degree of sequence identity.

**Figure 5:**
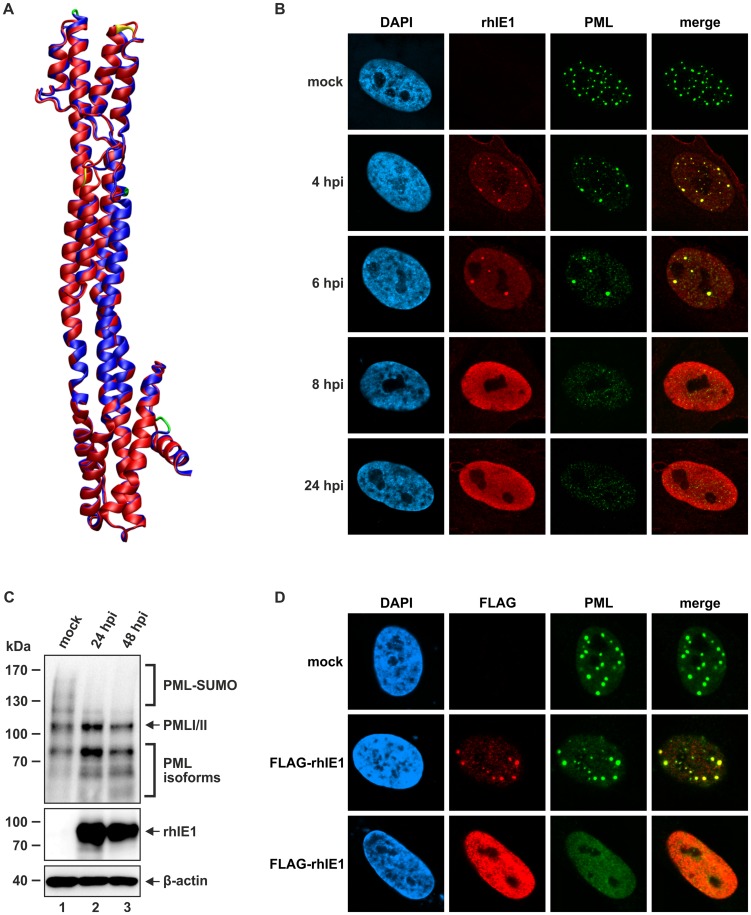
Structural and functional similarity between hIE1 and rhIE1. (A) Homology model of hIE1 (blue) superimposed onto the rhIE1 template crystal structure (red). The insertions (green) and deletions (yellow) of hIE1 compared to rhIE1 are mainly located in loop regions and do therefore not disrupt the secondary structure elements. Structure validation using ProSA indicates that the model exhibits a good global and local quality and the respective structural parameters are close to that of the template crystal structure (Figure S7) suggesting that hIE1 and rhIE1 adopt a highly similar fold. (B) Dispersal of PML-NBs after RhCMV infection of primary human fibroblasts (HFFs). HFF cells were infected with RhCMV at an MOI of 0.1 and harvested at indicated times for immunofluorescence analysis of rhIE1 and endogenous PML. Cell nuclei were counterstained with DAPI. (C) DeSUMOylation of PML after RhCMV infection of HFF cells. Cell lysates harvested either from mock infected cells or from cells infected with RhCMV at an MOI of 3 for indicated times (24, 48 h) were separated by SDS-PAGE and analyzed by Western blotting for expression of PML (upper panel), rhIE1 (middle panel) and β-actin as internal loading control (lower panel). (D) Dispersal of PML-NBs after transient expression of rhIE1 in HFFs. HFFs were transfected with a eukaryotic expression vector encoding rhIE1 fused to an N-terminal FLAG-tag and, after 48 h, subjected to indirect immunofluorescence analysis of rhIE1 using an anti-FLAG antibody and of endogenous PML; cell nuclei were stained with DAPI. Upper panel: mock transfected cells. Middle panel: colocalization of rhIE1 and PML, as observed in only few transfected cells. Lower panel: dispersed pattern of rhIE1 and PML, as observed in the majority of transfected cells.

This is in line with the results of a molecular model generated for hIE1 based on the rhIE1 crystal structure (Figure 5A). This model exhibits a good global and local quality further indicating that hIE1_CORE_ and rhIE1_CORE_ adopt highly similar folds (Figure S7). Due to the observed structural conservation we asked whether rhIE1 is likewise capable of disrupting human PML-NBs. Infection of primary human fibroblasts with rhesus macaque cytomegalovirus (RhCMV) revealed an initial accumulation of rhIE1 at PML-NBs followed by a dispersal of NBs (Figure 5B). Furthermore, RhCMV infection resulted in a depletion of polySUMOylated PML species (Figure 5C) and the isolated rhIE1 expression was sufficient to redistribute PML (Figure 5D, lower panel) indicating that hIE1 and rhIE1 do not only share biophysical properties but also functional activities.

### IE1_CORE_ induces deSUMOylation of PML but fails to disperse PML accumulations

**Figure 6:**
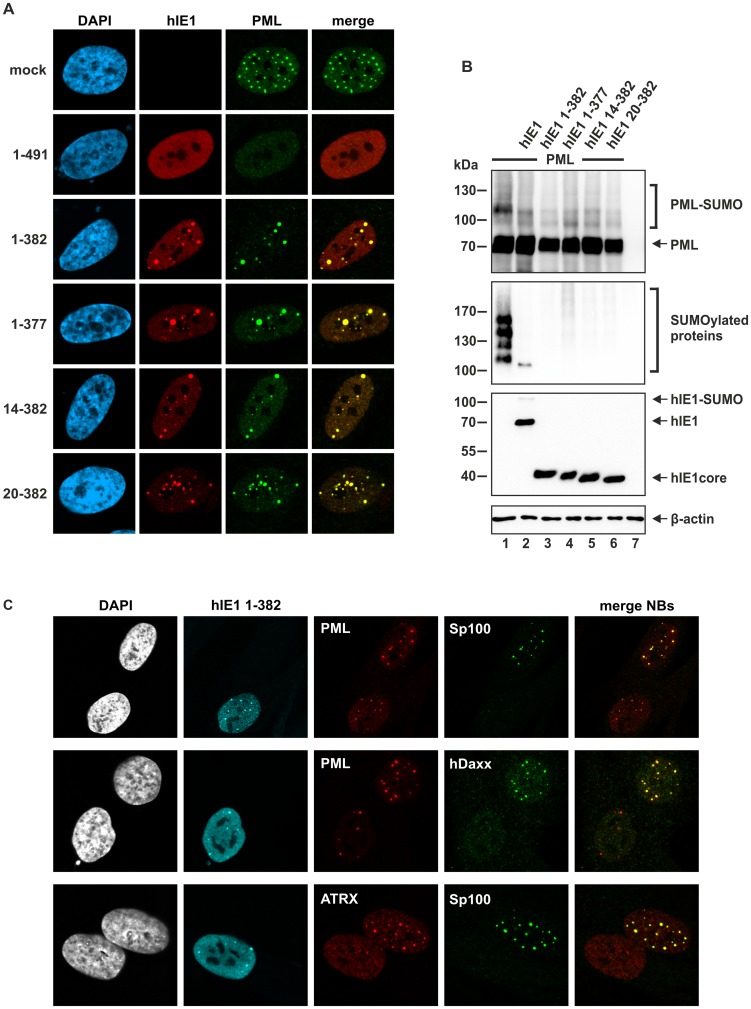
Effect of IE1_CORE_ on the integrity of PML-NBs and the SUMOylation of PML. (A) Dispersal of PML requires the IE1_IDRc_. HFF cells were transiently transfected with expression plasmids encoding FLAG-tagged full-length hIE1(1–491) or truncated hIE1 variants followed by immunodetection of hIE1 proteins using an anti-FLAG antibody and of endogenous PML. (B) DeSUMOylation of PML by IE1_CORE_. HEK293T cells were cotransfected with expression plasmids encoding FLAG-PML (isoform VI), FLAG-hIE1 variants as indicated and Myc-SUMO3. PML (upper panel), SUMOylated proteins (second panel), IE1 (third panel) and β-actin (lower panel) were detected by Western blot analysis. (C) Release of Sp100, hDaxx and ATRX from PML foci by IE1_CORE_. The hIE1_CORE_(1–382) was stably expressed in HFFs followed by immunodetection of hIE1, PML, Sp100, hDaxx or ATRX by triple staining as indicated.

In order to study whether the function of hIE1_CORE_ differs from that of full-length hIE1, we investigated the subcellular localization of the hIE1_CORE_ (Figure 6A). For this purpose, primary human fibroblast cells (HFFs) were transfected with eukaryotic expression plasmids encoding full-length hIE1 or truncated hIE1_CORE_ proteins. Surprisingly, while full-length hIE1 exhibited a dispersed nuclear localization and induced a loss of PML-aggregates, all truncated proteins showed a punctate staining pattern colocalizing with PML foci. Based on these data, we conclude that a region within the C-terminal hIE1_IDR_ is necessary for PML-dispersal. In contrast, hIE1_CORE_ alone was sufficient to induce deSUMOylation of PML in transient expression experiments using 293T cells (Figure 6B). Consistent results were obtained with a whole cell population of HFF cells stably expressing the hIE1_CORE_ variant 1–382 (Figure 6C). Given that SUMO modification of PML is a prerequisite for the recruitment of other NB components like Sp100, hDaxx and ATRX, it was important to explore the subcellular localization of these factors after expression of hIE1_CORE_. Interestingly, while PML was detected in a dot-like pattern, Sp100, hDaxx and ATRX were released from NBs in the presence of hIE1_CORE_ (Figure 6C). Taken together, these data demonstrate that hIE1_CORE_ is sufficient to sequester and deSUMOylate PML resulting in the dissociation of other NB components.

### IE1_CORE_ efficiently binds to PML via the coiled-coil domain

**Figure 7:**
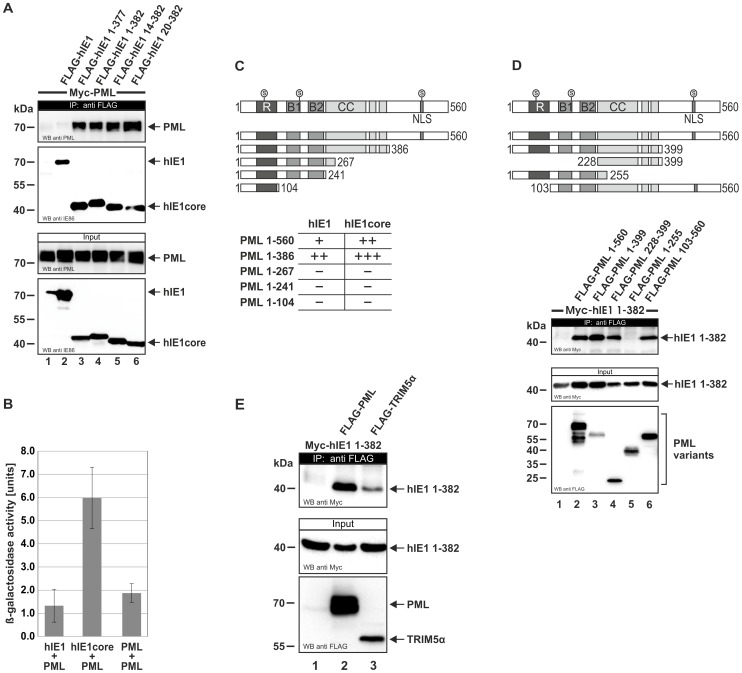
Interaction of IE1_CORE_ with PML and TRIM5α. (A) Enhanced binding of IE1_CORE_ to PML in coimmunoprecipitation analysis. HEK293T cells were cotransfected with expression plasmids encoding FLAG-hIE1 variants (as indicated) and Myc-PML (isoform VI). Upper two panels: Western blot detection of PML and hIE1 after immunoprecipitation with an anti-FLAG antibody. Lower two panels: detection of PML and hIE1 in cell lysates before precipitation (input). (B) Enhanced binding of IE1_CORE_ to PML in yeast two-hybrid assays. The graph shows the quantification of β-galactosidase reporter activity after coexpression of PML and full-length hIE1 or the hIE1_CORE_(14–382) in yeast cells. (C) The PML coiled-coil is required for IE1 binding in yeast two-hybrid assays. Full-length hIE1 and hIE1_CORE_(14–382) were tested for interaction with various C-terminal PML deletion mutants (shown in the upper scheme) by XGal filter lift assays. (D) The PML coiled-coil is sufficient for binding to hIE1_CORE_ in coimmunoprecipitation analysis. HEK293T cells were transfected with various FLAG-tagged PML deletion mutants, as shown in the upper scheme, together with Myc-hIE1_CORE_(1–382). Upper panel: Western blot detection of hIE1_CORE_ after immunoprecipitation with an anti-FLAG antibody. Lower two panels: Western blot detection of PML variants and hIE1_CORE_ in cell lysates before precipitation (input). (E) Interaction of hIE1_CORE_ with TRIM5α. HEK293T cells were cotransfected with expression plasmids encoding Myc-tagged hIE1_CORE_(1–382) and either FLAG-tagged PML (isoform VI) or FLAG-tagged rhesus macaque TRIM5α (rhTRIM5α). Upper panel: Western blot detection of hIE1_CORE_ after coimmunoprecipitation using an anti-FLAG antibody. Lower two panels: Western blot detection of PML, rhTRIM5α and hIE1_CORE_ in cell lysates before precipitation (input). R, RING domain; B, B-boxes; CC, coiled-coil domain; NLS, nuclear localization signal; S, SUMO.

Due to the accumulation of hIE1_CORE_ at PML foci, it was attractive to speculate that the two proteins might strongly interact with each other, which was investigated by co-immunoprecipitation (Figure 7A). Intriguingly, while only a trace amount of PML was associated with full-length hIE1, PML was efficiently coprecipitated with hIE1_CORE_ variants. An increased affinity of hIE1_CORE_ for PML was also confirmed by yeast two-hybrid experiments (Figure 7B), which is in line with previous results by Lee et al. (2004) that show an enhanced interaction of PML with an IE1 variant lacking the acidic C-terminus (IE1 1–420) [35]. Having observed a structural similarity between IE1 and coiled-coil regions of TRIM proteins, we asked whether this domain of PML is required for binding of IE1. In a yeast two-hybrid analysis utilizing a series of C-terminal PML deletion mutants we observed that a truncation of the coiled-coil domain abrogated the interaction with IE1 (Figure 7C). To further confirm this finding, coimmunoprecipitation analyses were performed with additional N- and/or C-terminal PML deletions. Importantly, this experiment revealed that the coiled-coil domain of PML was sufficient to mediate an interaction with IE1 (Figure 7D, lower panel, lane 4). Furthermore, we observed that IE1 was also able to bind to TRIM5αsuggesting that IE1 targets additional TRIM factors via coiled-coil interactions (Figure 7E).

### IE1_CORE_ antagonizes PML-mediated intrinsic immunity during viral infection

**Figure 8:**
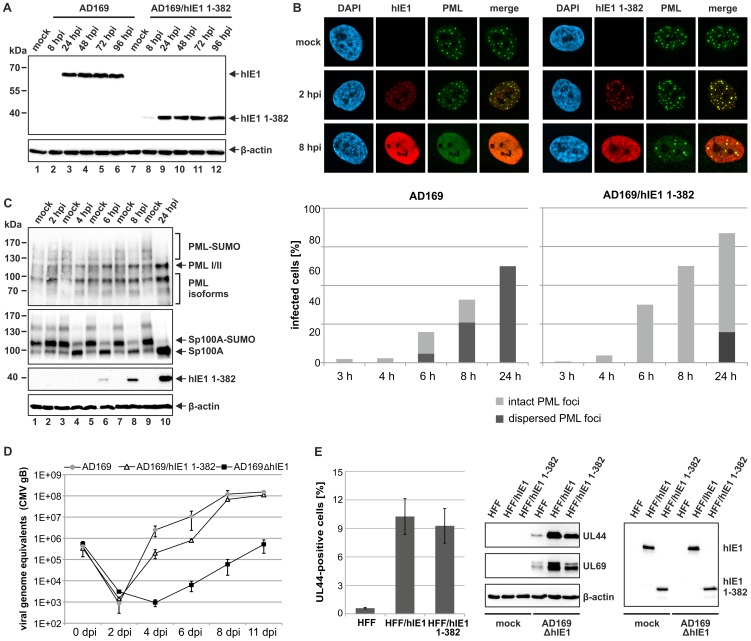
Analysis of IE1_CORE_ during human cytomegalovirus replication. (A) Western blot analysis showing the expression kinetics of full-length hIE1 or hIE1_CORE_(1–382) after infection of HFFs with wild-type HCMV (AD169) or recombinant HCMV expressing truncated hIE1 (AD169/hIE1 1–382) (MOI 0.2). (B) PML-NB dispersal after infection of HFFs with either AD169 or AD169/hIE1 1–382. Upper panel: immunofluorescence detection of hIE1 and PML at 2 and 8 hpi with the respective viruses (MOI 0.5). Lower graph: quantification of the number of infected cells containing either intact or dispersed PML foci during the first 24 h after infection. (C) DeSUMOylation of PML and Sp100 after infection of HFFs with AD169/hIE1 1–382 (MOI 3). Western blot detection of PML and Sp100 (upper panels) as well as hIE1 1–382 and β-actin (lower panels) during the first 24 hpi. (D) Growth curve analysis of wild-type AD169, AD169/hIE1 1–382 and IE1-deleted HCMV (AD169ΔhIE1). HFF cells were infected with wild-type AD169 at a low MOI (MOI 0.01) and equivalent genome copies of AD169/hIE1 1–382 and AD169ΔhIE1. Cell supernatants were harvested at indicated times and analyzed for genome copy numbers by quantitative real-time PCR. (E) Complementation of AD169ΔhIE1 by hIE1 1–382. Left panel: HFFs or HFFs expressing either hIE1 or hIE1 1–382 were infected with AD169ΔhIE1 at a low MOI (MOI 0.01). The cells were fixed at 48 hpi followed by immunofluorescence staining of UL44 and quantification of cells exhibiting viral gene expression using the ImageJ software. Middle and right panel: Western blot analysis of UL44, UL69, β-actin and IE1 expression in HFF, HFF/hIE1 or HFF/hIE1 1–382 cells after either mock infection or infection of with AD169ΔhIE1 as indicated.

Having shown that hIE1_CORE_ binds with high affinity to and deSUMOylates PML, but fails to disrupt PML accumulations, it was important to investigate whether this is sufficient to antagonize PML-NB mediated repression of viral infection. We constructed a recombinant HCMV expressing hIE1 lacking the C-terminal IE1_IDR_ (Figure 8A) and could observe that this virus exhibited a severe defect to disperse PML after infection of HFFs (Figure 8B). Consistent with our results obtained after isolated expression of hIE1_CORE_, deSUMOylation of both PML and Sp100 was fully preserved (Figure 8C). Most importantly, however, the hIE1_CORE_-expressing virus replicated nearly as efficient as wild-type virus while an hIE1-deleted virus exhibited a severe growth defect (Figure 8D). The approximately 10fold growth reduction observed for the hIE1_CORE_-expressing virus at 4 and 6 dpi is in line with previously published results on viruses lacking the C-terminal acidic domain which binds STAT2 thus antagonizing the interferon response [28], [29]. This was also confirmed in a complementation experiment after infection of either hIE1- or hIE1_CORE_-expressing HFFs with an hIE1-deleted HCMV, finally demonstrating that hIE1_CORE_ can efficiently substitute for full-length hIE1 during lytic HCMV infection (Figure 8E).

## Discussion

The immediate-early protein IE1 of human cytomegalovirus that directly binds to PML is known as an important herpesviral antagonist of PML-NB-mediated intrinsic immunity [15], [22], [33], [36]. However, the structural basis for its function has remained elusive due to the paucity of high-resolution structural information. Here, we present the first crystal structure for the evolutionary conserved primate cytomegalovirus IE1 proteins and demonstrate that a structurally conserved IE1_CORE_ domain is sufficient to antagonize PML-mediated intrinsic immunity. The structure of IE1_CORE_ consists of a femur-shaped bundle of helices, which surprisingly does not share any overall fold similarity with known protein structures. IE1_CORE_ binds with high affinity to PML and efficiently abrogates PML SUMOylation, but fails to disrupt PML accumulations itself. Only upon inclusion of the C-terminal, intrinsically disordered region (IE1_C-IDR_) PML dispersal is observed. Thus, our study demonstrates that PML deSUMOylation can be discriminated from PML dispersal. Whereas the first activity is achieved by a distinctly folded IE1_CORE_ domain, the second activity requires inclusion of C-terminal sequences of the IE1_C-IDR_ region that is highly susceptible to proteolytic degradation and for which we did not observe any stable secondary structure formation. As it has been observed for many intrinsically disordered proteins, folding of the natively disordered IE1_C-IDR_ region may occur upon binding to a specific interaction partner. First evidence for this comes from a recent study predicting that the chromatin-tethering domain (CTD) at the extreme C-terminus of IE1 forms a β-hairpin when bound to histone proteins [47]. Importantly, IE1_CORE_ is able to release other NB-components like Sp100, hDaxx and ATRX into the nucleoplasm and this correlates with antagonization of NB-mediated repression. This shows that PML dispersal as observed during infection with herpes simplex virus type I and HCMV is not a prerequisite to antagonize the repressive effects of this cellular multiprotein complex on viral gene expression [24], [48]. As also suggested by recent findings on the γ-herpesviruses Herpesvirus *saimiri* and Kaposi sarcoma herpesvirus as well as on the polyomavirus BKV more subtle modifications like the release or degradation of individual NB-components appear to be sufficient [49]–[51].

IE1_CORE_ resembles the action of the SUMO-specific protease SENP-1 which also abrogates the SUMOylation of PML but leaves most PML aggregated [52]. It was previously speculated that IE1 could harbor an intrinsic SUMO protease activity itself or recruit SUMO-specific proteases to the NBs [53]. However, our structural analysis of IE1_CORE_ provides no evidence for the presence of a potential active site with hydrolase activity. Furthermore, in earlier studies no interaction of IE1 with SENPs could be detected, but it was reported that full-length hIE1 could still disassemble foci formed by a PML protein with all SUMOylation sites mutated [53]. Based on this observation, the authors raised the idea that SUMO-independent interference with PML oligomerization followed by exposure of SUMOylated PML to cellular SUMO proteases may account for NB disruption. However, the results of our study argue against such a scenario, since abrogation of PML SUMOylation by IE1_CORE_ was detected while PML aggregates were still present. Thus, IE1 affects its targets via direct, SUMO-independent substrate interaction and this suggests that IE1 does not directly or indirectly act as a hydrolase that specifically targets SUMOylated PML.

Importantly, our study revealed structural similarities between IE1_CORE_ and the crystal structure of the tripartite motif coiled-coil that appears to act as a critical scaffold organizing the biochemical activities of TRIM proteins [44], [45]. Moreover, we were able to confirm that the coiled-coil of PML is sufficient for strong binding to IE1. Increasing evidence suggests that TRIM proteins function as E3-ubiquitin ligases in agreement with the family-wide presence of several conserved domains, namely a RING domain followed by two B-boxes and a coiled-coil region [3]. Based on the recently solved crystal structure of the TRIM25 coiled-coil it was shown that TRIM proteins dimerize by forming interdigitating antiparallel helical hairpins that position the N-terminal catalytic RING domain at opposite ends of the dimer and the C-terminal substrate-binding domains at the center [44]. For some of the TRIM members, and among these PML, E3-SUMO instead of E3-ubiquitin ligase activity has been reported [5]. Thus, we would like to propose that IE1_CORE_, via its strong interaction with the PML coiled-coil, may inhibit an E3-SUMO ligase activity of PML that is required for auto-SUMOylation. Alternatively, IE1 binding to the coiled-coil might block the accessibility of PML for other components of the cellular SUMOylation machinery. Thus, the results of our study favor a model whereby IE1 primarily affects the on-rate of SUMO modification which is also supported by the slow kinetics of IE1-mediated loss of PML SUMOylation [54]. This is different from the ICP0 protein of herpes simplex virus type I which induces the rapid degradation of SUMO-conjugated proteins by acting as a SUMO-targeted ubiquitin ligase (STUbL) [21]. Similar to IE1, the adenoviral E4-ORF3 protein which has been shown to form a multivalent matrix via extensive self-interactions, appears to inactivate PML via tight binding [55]. This specific assembly of E4-ORF3 creates avidity-driven interactions that capture PML as well as other tumor suppressors thus disrupting PML bodies. However, in contrast to IE1, the recently solved crystal structure of E4-ORF3 revealed the molecular mechanism of multimerization, but not the exact mode of PML recognition [55].

In this context it should be noted that the nonstructural NS1 protein of influenza A virus has also been shown to target a TRIM protein, TRIM25, via interaction with the coiled-coil domain to inhibit its E3 ligase function [56]. Since TRIM25 catalyzes a critical ubiquitination of the viral RNA sensor RIG-I this constitutes a mechanism by which influenza virus inhibits the host IFN response. Interestingly, we detected that IE1_CORE_ not only binds to PML but also to TRIM5α and a recent publication reported an interaction with TRIM33 [57]. Thus, the unique structure of IE1core may have developed during evolution to target an extended spectrum of members of the TRIM family via the conserved coiled-coil domain of these factors [44], [45]. This is also supported by our analysis of evolutionary conserved surface patches of IE1_CORE_. When assuming that sites of protein-protein interaction are reflected by conserved surface patches, our observation that conserved residues are distributed evenly over the entire IE1_CORE_ protein surface suggests that rather large parts of the IE1 surface are involved in recognition of the PML coiled-coil. Consequently, the helical structure of IE1_CORE_ might have evolved as a decoy that, by means of extensive helix-helix interactions might either pair up with the coiled-coil region of PML or substitute for one of the PML monomers within the PML dimer interface. In this respect, the similarity between the topological arrangement of helices H1 to H3 of IE1 and of predicted helices H3 to H1 of PML in combination with the joint occurrence of regions with extended hendecad repeats might facilitate the formation of heteromeric assemblies. The formation of extended coiled-coil interactions would also readily offer an explanation for the finding that single mutations within the conserved surface patches of IE1 only moderately affect its interaction properties with PML. In contrast, mutations affecting the overall tertiary structure (e.g. IE1 L174P) abrogate the functionality.

Furthermore, it agrees with our observation that rhIE1 can substitute for hIE1 during infection of human cells despite low overall sequence identity. Thus, the size and unique elongated fold of the IE1_CORE_ could have developed during evolution to accommodate efficient binding of PML and possibly other TRIM factors *via* an extended surface involving coiled-coil interactions. This feature might render the interaction less amenable to mutational escape.

## Materials and Methods

### Recombinant protein production and purification

All variants of h-, c- and rhIE1 were recombinantly produced in *E.coli* strain BL21(DE3) (Novagen) as GST-tagged fusion proteins for *in vitro* experiments and crystallization. LB media (Carl Roth GmbH + Co. KG, Karlsruhe, Germany) were inoculated with freshly transformed *E. coli* colonies, and cell cultures grown at either 30° or 37°C. Seleno-methionine labeling of rhIE1(residues 36–395) was achieved by incubation of the cells with non-inducing PAG medium (pre-culture) and auto-inducing PASM-5052 medium (main culture). Cell pellets were resuspended in phosphate buffer and lysed by sonication. Protein purification was achieved by the following steps: a first affinity chromatography (Glutathione sepharose, GE Healthcare, Freiburg), proteolytic cleavage with PreScission protease, a second affinity chromatography and a final size exclusion chromatography (Superdex 200 prepgrade, GE Healthcare). The gel filtration column was pre-equilibrated in 25 mM Tris, 150 mM NaCl, 10 mM DTT, pH 7.5. The samples were separated with an isocratic gradient of 1.2 column volumes (CV) of the same buffer at a flow rate of 1.5 mL/min. The column was calibrated utilizing the elution peaks of thyroglobulin (670 kDa), bovine γ-globulin (158 kDa), chicken ovalbumin (44 kDa) and equine myoglobin (17 kDa) of the Bio-Rad gel filtration standard (Bio-Rad Laboratories, Munich, Germany). The molecular weight of the samples was determined by linear regression. The Kav coefficients of the standard proteins were plotted vs the logarithm of their molecular weights to obtain the calibration curve, with Kav = (Ve-V0)/(Vc-V0), where V0 is the column void volume, Ve is the elution volume and Vc is the geometric column volume. All purification steps were performed in the presence of 10 mM DTT. For the crystallization of variant rhIE1(36–395) the protein was chemically modified by lysine methylation prior to the final size exclusion chromatography step. A 1 mg/mL IE1 protein solution was incubated on ice with 20 µL of 1 M dimethylamine borane (DMAB) and 40 µL of 1 M formalin per mL of IE1 solution. After two hours the addition of DMAB and formalin was repeated and, following an additional two-hour incubation, 10 µL of 1 M DMAB per mL of IE1 solution were added, and the solution was incubated at 4°C overnight. The reaction was quenched by adding 125 µL 1 M Tris/HCl, pH 7.5 per mL of IE1 solution, and the protein was stabilized by addition of 10 mM DTT [58]. Following the final chromatography step, the protein samples were concentrated to 20 mg/ml and stored at −20°C in 25 mM Tris/HCl, 1.5 mM NaCl, 15 mM DTT, 1 mM EDTA, pH 7.4 before further usage.

### Limited proteolysis

Limited proteolysis was performed in order to probe the conformational architecture of the protein [59]. The assay was conducted at 21°C with protein concentrations between 0.2 and 0.5 mg/mL and 0.014 mU subtilisin (Sigma-Aldrich) per µg IE1 protein. Aliquots of 10 µL were taken at different time points, for example at 1 min, 10 min, 30 min, 60 min, 120 min, 180 min, 240 min and 300 min, mixed with 3.3 µL 4× SDS loading buffer and boiled at 95°C for 5 min to stop the cleavage reaction.

### Circular dichroism spectroscopy

Circular dichroism spectra were recorded between 185 and 260 nm from protein samples containing 1.5 µM or 2 µM protein for full-length or truncated IE1, respectively. The measurements were performed in 10 mM potassium phosphate buffer, pH 7.5 with a Jasco J-815 spectropolarimeter (Jasco, Tokyo, Japan) at 20°C with standard sensitivity. The cuvette had a path length of 0.1 cm, the band width was 1.0 nm, the scan speed 20 nm*sec^−1^, data integration time 1 sec and the data pitch 0.1 nm. All measurements were accumulated ten times and corrected for the sample buffer. Conversion of the data to concentration-independent mean residual weight (MRW) ellipticities [θ]_MRW_ was done as described previously [40].

### Crystallization of rhIE1(36–395)

Initial crystallization conditions were identified with a sparse matrix screening approach (Index Screen, Hampton Research, Aliso Viejo, USA) and a Phoenix protein crystallization robot (Art Robbins Instruments, Sunnyvale, USA) [60]. The crystallization conditions were optimized in a hanging drop vapor diffusion setup and involved microseeding. Crystals of diffraction quality were obtained by mixing 1 µL of protein solution with 1 µL of reservoir solution and equilibrating the droplet of 2 µL against 700 µL reservoir solution [0.4 M magnesium formate, 15% (w/v) PEG 3350]. The crystals were soaked in cryo-solution [0.4 M magnesium formate, 15% (w/v) PEG 3350, 15% (v/v) ethylenglycol or 20% (v/v) dimethyl sulfoxide (DMSO)] prior to flash-cooling in liquid nitrogen.

### Crystallographic data collection and crystal dehydration

The crystal structure of rhIE1(36–395) was initially solved in space group P2_1_ using the following diffraction datasets collected at 100 K at beamline BL14.1 at BESSY II synchrotron (Helmholtz Zentrum Berlin): a native dataset 1 extending to 2.85 Å resolution, a MAD dataset (peak, inflection point, remote) recorded from a gold-soaked crystal diffracting to 3.5 Å and a peak dataset from a seleno-methionine derivatized protein crystal diffracting to 3.1 Å resolution (Table S1). The gold-soaked crystal was prepared by incubating crystals for three days in cryo-solution containing DMSO and 2.5 mM KAu(CN)_2_. Before flash cooling, the crystals were back-soaked in cryo-solution for several minutes to remove unspecifically bound heavy atoms. All monoclinic datasets are highly isomorphous. The Matthews coefficient is 2.96 (58.42% solvent) when assuming the presence of four rhIE1(36–395) molecules in the asymmetric unit [61]. The final refinement of rhIE1(36–395) was performed against a dataset with space group symmetry P4_3_ extending to 2.3 Å (Table S1). The increase in resolution and concomitant space group change were obtained upon controlled dehydration of the monoclinic crystals from above with the HC1c crystal humidifier device at beamline BL14.3 at BESSY II (Helmholtz Zentrum Berlin). Crystals were first equilibrated against 98% relative humidity before decreasing the humidity in steps of 4% and 10 min equilibration time to a final value of 86% humidity. Upon observation of an increase in diffraction power, the crystals were flash cooled and transferred to beamline BL14.1 for the recording of a complete dataset extending to 2.3 Å resolution (Table S1). All diffraction datasets were processed with XDS and scaled with XSCALE [62].

### Structure determination and refinement

Initial protein phases were derived for the monoclinic crystal form using the MAD dataset collected from a gold-soaked crystal (Table S1). The positions of the gold atoms could be readily located with program SHELXD [63]. The non-crystallographic symmetry (NCS) relationship between the 4 monomers, i.e. the presence of two IE1 dimers with C2 point group symmetry, became apparent upon visualization of the gold positions in program COOT and the inspection of the initial electron density maps calculated with program SHELXE [64], [65]. The NCS relationship was corroborated by the self-rotation function, calculated with program POLARRFN from the CCP4 program suite [66]. The quality of these initial electron density maps could be significantly improved upon phase calculation with program SHARP/autoSHARP [67], [68] and density averaging with program DM [69]. The improved phases also allowed for the identification of the selenium positions in the peak dataset of the seleno-methionine-labeled protein crystal and the inclusion of this dataset in the calculation of the experimental protein phases. An initial atomic model covering a single monomer was then manually built starting from protein fragments derived with program autoSHARP and following the lead of electron density maps calculated with either program autoSHARP or MLPHARE/DM [66], [68]. The registration of the protein sequence was obtained from the shape of the local electron density and the positions of the selenium atoms as visualized by an anomalous difference map. These considerations also showed that one gold cation is bound *via* a free cysteine side chain in each IE1 monomer chain. The model was then stepwise completed, extended to four molecules in the asymmetric unit and refined with program PHENIX [70]. Convergence of the refinement at 2.8 Å in space group P2_1_ was facilitated upon inclusion of NCS weights and secondary structure restraints in program PHENIX [70]. The final model of rhIE1(36–395) was obtained after transferring the monoclinic model into the tetragonal unit cell with program PHASER and upon refinement against the 2.3 Å dataset in space group P4_3_ (Table S1) [71]. Refinement converged at crystallographic R-factors of 19.73% (R_work_) and 24.96% (R_free_). The space group change that took place upon dehydration can be easily explained by small readjustments in the packing of the IE1 dimers in the crystals.

### PDB accession numbers

Coordinates and structure factors for the IE1_CORE_ structure have been deposited in the Protein Data Bank under accession code 4WID.

### Bioinformatic analyses

The size of the dimerization interface between the two rhIE1_CORE_ monomers was calculated with the program PISA [72]. The reported value is the average of the buried surface of both chains. The rhIE1_CORE_ monomers shown in Figure S5 were superposed with the program LSQKAB [66]. Only the α-helical segments of the protein as defined in Figure S1 were superimposed. Comparative modeling of hIE1 was performed with MODELLER 9.9 [73] and the resulting model was validated using ProSA [74], [75]. Searches for structurally similar proteins were performed with PDBeFold [76]. Since standard parameters did not result in any hits, the following search options were set. (i) The threshold of 70% for the lowest acceptable match in query and target was reduced to 30% and 60%, respectively. (ii) The search was extended to proteins with a different connectivity of their secondary structure elements. Searches were performed independently for chain A and chain B of rhIE1, and the list of hits was merged. For reasons of clarity, duplicate hits and hits related closely in sequence (>90% identity) were removed from the list. The normRMSD was calculated according to the following equation [77]: normRMSD  =  [RMSD • max(L_1_,L_2_)]/N_aln._ Where RMSD is the root mean square deviation of the superposition of query and target, max(L_1_,L_2_) is the number of amino acids of the largest chain in the superposition, and N_aln_ is defined by the number of structurally equivalent residue pairs. Sequence conservation was calculated based on a Blosum30 matrix using the MultiSeq Plugin [78] of VMD [79]. The IE1 sequences from the following viruses served as input: human CMV (strain AD169), Rhesus-CMV, Baboon-CMV, Simian-CMV, and Panine-HV2/Chimpanzee CMV (Uniprot-accession-numbers P13202, Q2FAE9, D0UZW7, Q98682, Q8QRY6). Prediction of intrinsically disordered regions in hIE1, cIE1 and rhIE1 was performed with IUPred [39] using the prediction type “short disorder”. The disorder tendencies in the three IE1 homologs were plotted in one diagram using the rhIE1 amino acid numbering. Multiple sequence alignment was performed with TCoffee (http://www.ebi.ac.uk/Tools/services/web/toolform.ebi?tool=tcoffee).

### Oligonucleotides and plasmid constructs

The oligonucleotide primers used for this study were purchased from Biomers GmbH (Ulm, Germany) and are listed in Table S3. All prokaryotic expression plasmids were generated by PCR amplification of the respective codon-optimized IE1 sequences and subsequent cloning into pGEX-6P-1 (GE Healthcare Bio-Sciences AB, Uppsala, Sweden). The synthetic, codon-optimized hIE1 cDNA (strain AD169) was obtained from Mr. Gene GmbH (Regensburg, Germany). The codon-optimized cDNAs of cIE1 (NP_612746.1) and rhIE1 (Q2FAE9) were synthesized by GENEART gene synthesis service (Regensburg, Germany). The eukaryotic expression plasmids encoding full length or truncated hIE1 were generated via PCR amplification of the respective fragments using pHM494 [80] as template, followed by insertion into pHM971 (pcDNA3.1-FLAG) [80], pHM1580 (pcDNA3.1-Myc) [80], or into the yeast expression vectors pGBT9 and pGAD424 (Clontech, Mountain View, CA). The synthetic gene coding for rhIE1 (Q2FAE9) was obtained from GENEART gene synthesis service (Regensburg, Germany). The rhIE1 coding sequence was subcloned into pHM971 (pcDNA3.1-FLAG) [80] using *Bam*HI and *Xho*I. Full length PML, isoform VI, and truncated PML variants were amplified from pAS-PML (a gift from G.G. Maul, Philadelphia, USA) and inserted into pHM1580 (pcDNA3.1-Myc) [81], pHM971 (pcDNA3.1-FLAG) [80], pHM972 (pcDNA3.1-FLAG-NLS), or yeast expression vectors pGBT9 and pGAD424 (Clontech, Mountain View, CA). The eukaryotic expression plasmid encoding rhesus TRIM5α was a gift from T. Gramberg (Erlangen, Germany). For transduction experiments, hIE1 variants were amplified utilizing pHM494 [80] as template and inserted into a pLKO-based lentiviral vector (a gift of R. Everett, Glasgow, UK).

### Cells, infections and viruses

HEK293T cells and primary human foreskin fibroblast (HFF) cells (obtained from Life Technologies) or telomerase-immortalized HFFs (HFFi) were cultured as described previously [54], [82]. HFFs were infected with either the HCMV laboratory strain AD169, a recombinant HCMV expressing hIE1 1–382 (AD169/hIE1 1–382), an IE1-deficient virus (AD169ΔhIE1), or rhesus macaque CMV (RhCMV) at specified multiplicities of infection (MOI). Titers of wild-type (wt) AD169 and the AD169 recombinants were determined by UL112/113 fluorescence. For this purpose, HFFs were infected with various dilutions of virus stocks. After 72 h of incubation, cells were fixed and stained with a monoclonal antibody directed against UL112/113. Subsequently, the number of UL112/113-positive cells was determined and was used to calculate viral titers. RhCMV was titrated via rhIE1 fluorescence, which was analyzed 24 h postinfection.

The AD169-based HCMV bacterial artificial chromosome (BAC) HB15 was used for recombination-based genetic engineering of AD169/hIE1 1–382 and AD169ΔhIE1. AD169/hIE1 1–382 was constructed by introducing a stop codon into the hIE1 gene replacing residue 383. For this purpose, the two-step red-mediated recombination technique was utilized [83], which uses the kanamycin gene as a first selection marker. The linear recombination fragment was generated by PCR using primers 5′BAC_short and 3'BAC_hIE1_382 (Table S3), and pEPkan-S (kindly provided by K. Osterrieder, Berlin) as template DNA. The PCR product was treated with DpnI, gel purified, and subjected to a second round of PCR amplification using primers 5′BAC_hIE1_382 and 3′BAC_hIE1_382_short (Table S3). For homologous recombination, the PCR fragment was transformed into *Escherichia coli* strain GS1783 (a gift of M. Mach, Erlangen) already harboring HB15, and bacteriophage λ red-mediated recombination was conducted as described elsewhere [83]. To identify positive transformants, the bacteria were plated on agar plates containing 30 µg/mL kanamycin (first recombination) or 30 µg/mL chloramphenicol and 1% arabinose (second recombination) and incubated at 32°C for 2 days. BAC DNA was purified from bacterial colonies growing on these plates and was further analyzed by PCR, restriction enzyme digestion and direct sequencing. For construction of the AD169ΔhIE1 BAC by homologous recombination, a linear recombination fragment, comprising a kanamycin resistance marker along with 5′ and 3′ genomic sequences, was generated by PCR amplification using pKD13 as template and primers 5′Intron3/pKD13 and 3′Exon 4/pkd13 (Table S3). This fragment was used for electroporation of competent *Escherichia coli* strain DH10B harboring HB15 and recombination was performed as described previously in order to delete exon 4 of the IE1 gene [84]. The integrity of the resulting recombinant BAC was confirmed by PCR, restriction enzyme digestion and direct sequencing.

For reconstitution of recombinant AD169, HFFs seeded in six-well dishes (3×10^5^ cells/well) were cotransfected with 1 µg of purified BAC DNA, 0.5 µg of the pp71 expression plasmid pCB6-pp71, and 0.5 µg of a vector encoding the Cre recombinase using FuGENE6 transfection reagent (Promega, Mannheim, Germany). Transfected HFFs were propagated until viral plaques appeared, and the supernatants from these cultures were used for further virus propagation.

### Lentivirus transduction and selection of stably transduced cells

For the generation of HFF cells stably expressing full length hIE1 or hIE1 1–382, replication-deficient lentiviruses were generated using pLKO-based expression vectors. For this purpose, HEK293T cells seeded in 10 cm dishes (5×10^6^ cells/dish) were cotransfected with a pLKO vector encoding either full length hIE1 or hIE1 1–382 together with packaging plasmids pLP1, pLP2, and pLP/VSV-G using the Lipofectamine 2000 reagent (Invitrogen, Karlsruhe, Germany). Viral supernatants were harvested 48 h after transfection, cleared by centrifugation, filtered, and stored at −80°C. Primary HFFs or telomerase-immortalized HFFs were incubated for 24 h with lentivirus supernatants in the presence of 7.5 µg/mL polybrene (Sigma-Aldrich, Deisenhofen, Germany). Stably transduced cell populations were selected by adding 500 µg/mL geneticin to the cell culture medium.

### Transfection

HFF cells were transfected with the DNA transfection reagent FuGENE6 (Promega, Mannheim, Germany). One day before transfection, 3×10^5^ cells were seeded into six-well dishes. DNA content and transfection procedure were according to the instructions of the manufacturer. 48 hours after transfection, cells were harvested for further analyses. HEK293T cells were transfected by applying the standard calcium phosphate precipitation method. For this, 5×10^5^ to 5×10^6^ HEK293T cells were seeded into six-well dishes or 10 cm dishes one day before transfection. For Western blot analyses and coimmunoprecipitations, 1 to 10 µg of plasmid DNA were used for each transfection reaction. At about 16 hours later, the cells were washed two times with PBSo and provided with fresh medium. 48 hours after transfection, cells were harvested for further analyses.

### Antibodies

Monoclonal antibodies used for immunofluorescence and Western blot analyses were: α-IE1 CH443 (Santa Cruz Biotechnology, Santa Cruz, CA, USA), α-UL112/113 M23, α-UL44 BS510 (kindly provided by B. Plachter, Mainz, Germany), α-UL69 69–66, α-FLAG M2 (Sigma-Aldrich, Deisenhofen, Germany), α-Myc 9E10, α-β-actin AC-15 (Sigma-Aldrich), α-PML PG-M3 (Santa Cruz). Polyclonal antibodies used for immunofluorescence and Western blot analyses were: α-rhesus IE1 (a kind gift from M. Mach, Erlangen, Germany), α-PML #4 (a kind gift from P. Hemmerich, Jena, Germany), α-PML H238 (Santa Cruz), α-PML A301–167A (Bethyl Laboratories, Montgomery, TX, USA), α-PML A301–168A (Bethyl Laboratories), α-Sp100 #2 (a kind gift from P. Hemmerich, Jena, Germany), α-Sp100 GH3 (kindly provided by H. Will, Hamburg, Germany), α-hDaxx C-20 (Santa Cruz), α-ATRX H-300 (Santa Cruz). Secondary antibodies used for immunofluorescence and Western blot analyses were: Alexa Fluor 488-/555-/647-conjugated secondary antibodies for indirect immunofluorescence experiments were purchased from Molecular Probes (Karlsruhe, Germany), horseradish peroxidase-conjugated anti-mouse/-rabbit secondary antibodies for Western blot analyses were obtained from Dianova (Hamburg, Germany).

### Indirect immunofluorescence

HFF cells grown on coverslips in six-well dishes (3×10^5^ cells/well) were washed twice with PBSo at 48 hours after transfection or at various times after virus infection. Cells were fixed with a 4% paraformaldehyde solution for 10 min at room temperature (RT) and then washed for two times. Permeabilization of the cells was achieved by incubation with 0.2% Triton X-100 in PBSo on ice for 20 min. Cells were washed again with PBSo over a time period of 5 min and incubated with the appropriate primary antibody diluted in PBSo-1% FCS for 30 min at 37°C. Excessive antibodies were removed by washing four times with PBSo, followed by incubation with the corresponding fluorescence-coupled secondary antibody diluted in PBSo-1% FCS for 30 min at 37°C. The cells were mounted using the DAPI-containing Vectashield mounting medium (Alexis, Grünberg, Germany) and analyzed using a Leica TCS SP5 confocal microscope, with 488 nm, 543 nm, and 633 nm laser lines, scanning each channel separately under image capture conditions that eliminated channel overlap. The images were exported, processed with Adobe Photoshop CS5 and assembled using CorelDraw ×5. In order to quantify PML-NB disruption in infected HFFs, 150 cells were analyzed for the presence of PML dots.

### Immunoblotting

Lysates from transfected or infected cells were prepared in a sodium dodecyl sulfate-polyacrylamide gel electrophoresis (SDS-PAGE) loading buffer, separated on sodium dodecyl sulfate-containing 8 to 15% polyacrylamide gels, and transferred to nitrocellulose membranes. Chemiluminescence was detected according to the manufacturer's protocol (ECL Western blot detection kit; Amersham Pharmacia Biotech).

### Coimmunoprecipitation

Transfected HEK293T cells (1×10^6^ or 5×10^6^ were lysed for 20 to 40 min at 4°C in 800 µL of CoIP buffer (50 mM Tris-HCl [pH 8.0], 150 mM NaCl, 5 mM EDTA, 0.5% NP-40, 1 mM PMSF, 2 µg/mL of aprotinin, 2 µg/mL of leupeptin, and 2 µg/mL of pepstatin). After centrifugation, aliquots of each sample were taken as input controls and the remaining supernatant was incubated with anti-FLAG antibody M2 coupled to protein-A-sepharose beads for 2 h at 4°C. The sepharose beads were collected by centrifugation and washed five times in 1 mL CoIP buffer. Finally, the immunoprecipitated proteins were recovered by boiling in 4× SDS sample buffer and protein complexes were analyzed by SDS-PAGE and Western blotting.

### Yeast two-hybrid analysis

*Saccharomyces cerevisiae* Y153 was used in a two-hybrid system. Both the plasmid pGBT9 (Clontech, Mountain View, CA) encoding the GAL4-DB (Trp+) fusion and the plasmid pGAD424 (Clontech, Mountain View, CA) encoding the GAL4-A fusion (Leu+) were introduced into Y153 cells using a modified lithium acetate (LiAc) method. For this, cells were grown overnight in YAPD medium, pelleted and treated with LP-mix (40% w/v PEG 4000, 0.15 M LiAc, 10 mM Tris/HCL pH 7.5, 1 mM EDTA pH 8.0) and DMSO. Single-stranded carrier-DNA as well as both plasmids were added to the yeast cells. This step was followed by incubation at room temperature and subsequent incubation at 42°C. Thereafter, the cells were plated on minimal selection agar lacking Trp and Leu. For rapid *in situ* assays of *lac*Z expression from yeast colonies, an XGal filter assay was used. Nitrocellulose filters were laid onto the plate and allowed to wet completely, then lifted off the plate and placed in liquid nitrogen to permeabilize the cells. The filters were removed and placed cell side up in a petri dish containing Whatman Paper soaked with Z buffer containing β-Mercaptoethanol and XGal. The filters were incubated at 30°C and constantly analyzed for the development of a positive blue color. For quantitation of the ß-galactosidase activity in the yeast cells three colonies were picked and grown in medium also lacking Trp and Leu. The next day, the optical density was measured at 600 nm. After pelleting the culture, the cells were resuspended in Z buffer and permeabilized using chloroform and 0.1% SDS. The ß-galactosidase activity within the cells was assayed by the standard method using o-nitrophenyl-ß-D-galactopyranoside (ONPG) as substrate. The reaction was stopped by adding Na_2_CO_3_ and the absorbance was measured at OD_405_. The unit of ß-galactosidase was defined as (1.000×OD_405_)/(t×v×OD_600_) (t, reaction time [min]; v, culture volume [mL]). The ß-galactosidase activity for each sample was corrected for background by subtracting the signal of the empty vectors.

### Multistep growth curve analysis

HFF cells were seeded into six-well dishes at a density of 3×10^5^ cells/well and infected the following day with wt AD169 at an MOI of 0.01 and equivalent genome copies of AD169/hIE1 1–382 and AD169ΔhIE1. Triplicate samples of the infected cell supernatants were harvested at 2, 4, 6, 8 and 11 days after inoculation and subjected to lysis by proteinase K treatment. Thereafter, all samples were analyzed for the amount of genome copy numbers by quantitative real-time PCR (TaqMan-PCR) using an Applied Biosystems 7500 Real-Time PCR System (Applied Biosystems, Foster City, CA, USA) together with the corresponding software SDS (sequence detection system) [85]. For quantification of the viral DNA load, a sequence region within the gB gene locus was amplified using primers 5'gB_forw and 3'gB_rev along with the fluorescence labeled probe CMV gB FAM/TAMRA also directed against the gB gene region. In parallel, the cellular DNA amount was quantified using primers 5′Alb and 3′ Alb together with a fluorescence labeled probe, Alb FAM/TAMRA, specific for the cellular albumin gene. Real-time PCR was performed in 96-well plates being compatible with the ABI Prism sequence detector. For the determination of reference C_T_ values (cycle threshold), serial dilutions of the respective standards (10^7^–10^1^ DNA molecules of gB or albumin) were examined by PCR reactions in parallel. The 20 µL reaction mix contained 5 µL sample or standard DNA solution together with 10 µL 2× TaqMan PCR Mastermix (Applied Biosystems, Foster City, CA, USA), 1.5 µL of each primer (5 µM stock solution), 0.4 µL of probe (10 µM stock solution), and 1.6 µL of H_2_O. The thermal cycling conditions consisted of two initial steps of 2 min at 50°C and 10 min at 95°C followed by 40 amplification cycles (15 sec at 95°C, 1 min 60°C). The viral genome copy numbers and albumin copy numbers were subsequently calculated using the sample-specific C_T_ value when set into relation to the standard serial dilutions.

### Complementation assay

For analysis of complementation by immunofluorescence staining, control HFFi cells as well as HFFi cells expressing hIE1 or hIE1 1–382 were infected with AD169ΔhIE1 at an MOI of 0.01. Triplicate samples of infected cells were fixed at 48 h post infection and subjected to immunostaining of UL44. Images of approximately 500 cells per sample were taken and the number of UL44-positive cells was determined via measuring of mean gray values using the ImageJ software (version 1.47). For analysis of complementation by Western blotting, normal HFFi cells as well as HFFi cells expressing hIE1 or hIE1 1–382 were infected with AD169ΔhIE1 at an MOI of 0.05 in triplicate, and were harvested 72 h later for detection of UL44, UL69, IE1 and β-actin.

## Supporting Information

Figure S1 Sequence alignment of homologous IE1 proteins: hIE1 of HCMV (strain AD169, AC146999), cIE1 of chimpanzee CMV (panine herpesvirus 2, NC003521.1), and rhIE1 of rhesus macaque CMV (ceropithecine herpesvirus 8, DQ120516.1). Sequence conservation is indicated by gray shading (dark gray: fully conserved residues, middle gray: strongly similar residues, light gray: weakly similar residues). Residues as resolved by the rhIE1 crystal structure are marked by a black border. The positions of the α-helices within this region, which are numbered consecutively from the N- to the C-terminus, were determined on the basis of the structural data. The positions of hydrophobic amino acids within hendecad and heptad repeats of helices 1 and 3 are indicated below the amino acid sequence (a, d, h).
(TIF)

Figure S2 The CD spectra of full-length hIE1(1–491) and hIE1(14–382) are shown in blue and gold, respectively (A). The difference spectrum obtained by subtracting the data of hIE1(14–382) from hIE1(1–491) is shown in green (B). The difference spectrum exhibits strong random coil characteristics with its minimum at a wavelength around 200 nm and therefore confirms the intrinsically disordered nature of the terminal regions.
(TIF)

Figure S3 Stereo image of a representative section of the experimentally phased electron density map used for model building. Protein phases in space group P2_1_ were derived from a 3.5 Å MAD dataset collected from a gold-soaked crystal and combined with 3.1 Å data from a SeMet SAD dataset collected at the peak wave-length of Se (Table S1). Extensive four-fold non-crystallographic symmetry averaging was applied. The protein density (contoured at 1σ) is shown in blue and the anomalous density calculated from the anomalous difference signal in the SeMet peak dataset (contoured at 3σ) in orange. The helical structure of the protein is clearly visible and the positions of the Se-atoms coincide with the locations of the peaks in the anomalous density map.
(TIF)

Figure S4 Multiple sequence alignment of the coiled-coil region of different TRIM proteins. The a and d position of the heptad and hendecad repeats and the h position present only in the hendecad repeats are highlighted.
(TIF)

Figure S5 Stereorepresentation showing the superposition of the two monomers that are present in dimeric rhIE1_CORE_ in the final refined structure in space group P4_3_. When considering the Cα positions from the α-helical segments, the two monomers can be superimposed with an r.m.s. deviation of 2.13 Å. The area with the highest deviation, namely, the loop between helices H8 and H9, is marked by a red box (see also Figure S6). The molecules were superposed with the program LSQKAB [66].
(TIF)

Figure S6 Simulated annealed OMIT difference density map illustrating a difference in the kink of the loop formed between helices H8 and H9 in the two monomers (panel A and B) of dimeric rhIE1_CORE_ (see also Figure S5). The maps were generated with program PHENIX and residues Glu276 to Thr292 omitted during refinement and subsequent difference density calculation. The σ_A_-weighted mFo-DFc electron density is displayed at a 3σ level.
(TIF)

Figure S7 Structure validation of the hIE1 model and comparison of the structural parameters to the rhIE1 template crystal structure. (A, B) Overall structure quality as indicated by the Z-score. The Z-scores of rhIE1 (A) and hIE1 (B) are displayed in a plot that contains the Z-scores of all experimentally determined protein chains in the PDB. In this plot, groups of structures from different sources (X-ray, NMR) are distinguished by different colors. Z-scores for IE1 chain A and B are denoted by a dot and a rectangle symbol, respectively. Note that the Z-scores of the crystal structure and model are very similar and well within the range expected for protein structures of this size. (C)–(F) Local structure quality evidenced by a knowledge-based energy as a function of amino acid sequence position. The plots are smoothed by calculating the average energy over each 10- residue fragment (thin line) or 40-residue fragment (thick line) along the peptide chain. The latter plots are similar for rhIE1 (C, D) and hIE1 (E, F) and exhibit favorable negative energy values along the entire peptide chains. This indicates that the modeling procedure has not placed residues in an unfavorable environment.
(TIF)

Table S1 Data collection, phasing and refinement statistics.
(DOC)

Table S2 List of structurally similar proteins of IE1 sorted by their Z-score in descending order. For each hit the PDB code, the RMSD of the overlay, and the number of structurally equivalent residues (N_algn_) is given. Generally, the number of equivalent residues is rather low covering less than 50% of the total length of IE1. This demonstrates that the overall fold in IE1 is rather unique and there are only local structural similarities to other helical proteins. These local hits were further inspected based on their CATH-classification. Most of the hits from the present search are classified as α-helical orthogonal bundle (code 1.10.) or up-down bundle (code 1.20.). Comparison of the third number of the CATH-code shows that these proteins belong to at least 18 different folds indicating that IE1 cannot readily be assigned to any known topology. This is further corroborated by the calculation of a normalized rmsd (normRMSD): For all hits listed in Table S2 the normRMSD values are significantly larger than the threshold of 5 Å [77], which indicates that a protein is significantly structurally different from those proteins previously deposited in the PDB.
(DOC)

Table S3 List of oligonucleotides.
(DOC)
