# Supplementary figures and images for: Crystal Structure of Cytomegalovirus IE1 Protein Reveals Targeting of TRIM Family Member PML via Coiled-Coil Interactions
Source: PLoS Pathog. 2014 Nov 20;10(11):e1004512. doi: 10.1371/journal.ppat.1004512 (PMC4239116; doi:10.1371/journal.ppat.1004512)

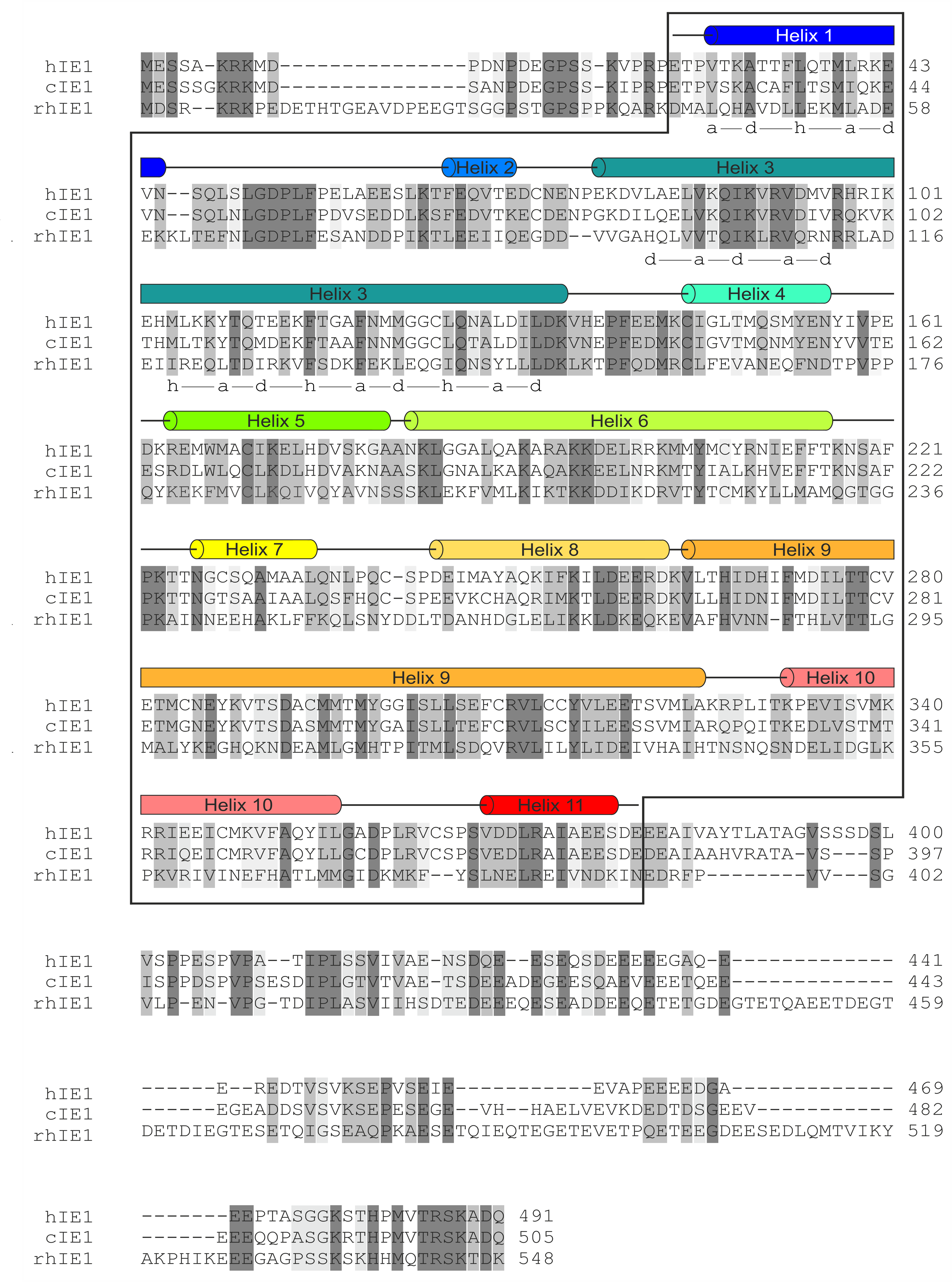

Supplement: Figure S1 — Sequence alignment of homologous IE1 proteins: hIE1 of HCMV (strain AD169, AC146999), cIE1 of chimpanzee CMV (panine herpesvirus 2, NC003521.1), and rhIE1 of rhesus macaque CMV (ceropithecine herpesvirus 8, DQ120516.1). Sequence conservation is indicated by gray shading (dark gray: fully conserved residues, middle gray: strongly similar residues, light gray: weakly similar residues). Residues as resolved by the rhIE1 crystal structure are marked by a black border. The positions of the α-helices within this region, which are numbered consecutively from the N- to the C-terminus, were determined on the basis of the structural data. The positions of hydrophobic amino acids within hendecad and heptad repeats of helices 1 and 3 are indicated below the amino acid sequence (a, d, h). (TIF) [file ppat.1004512.s001.tif]

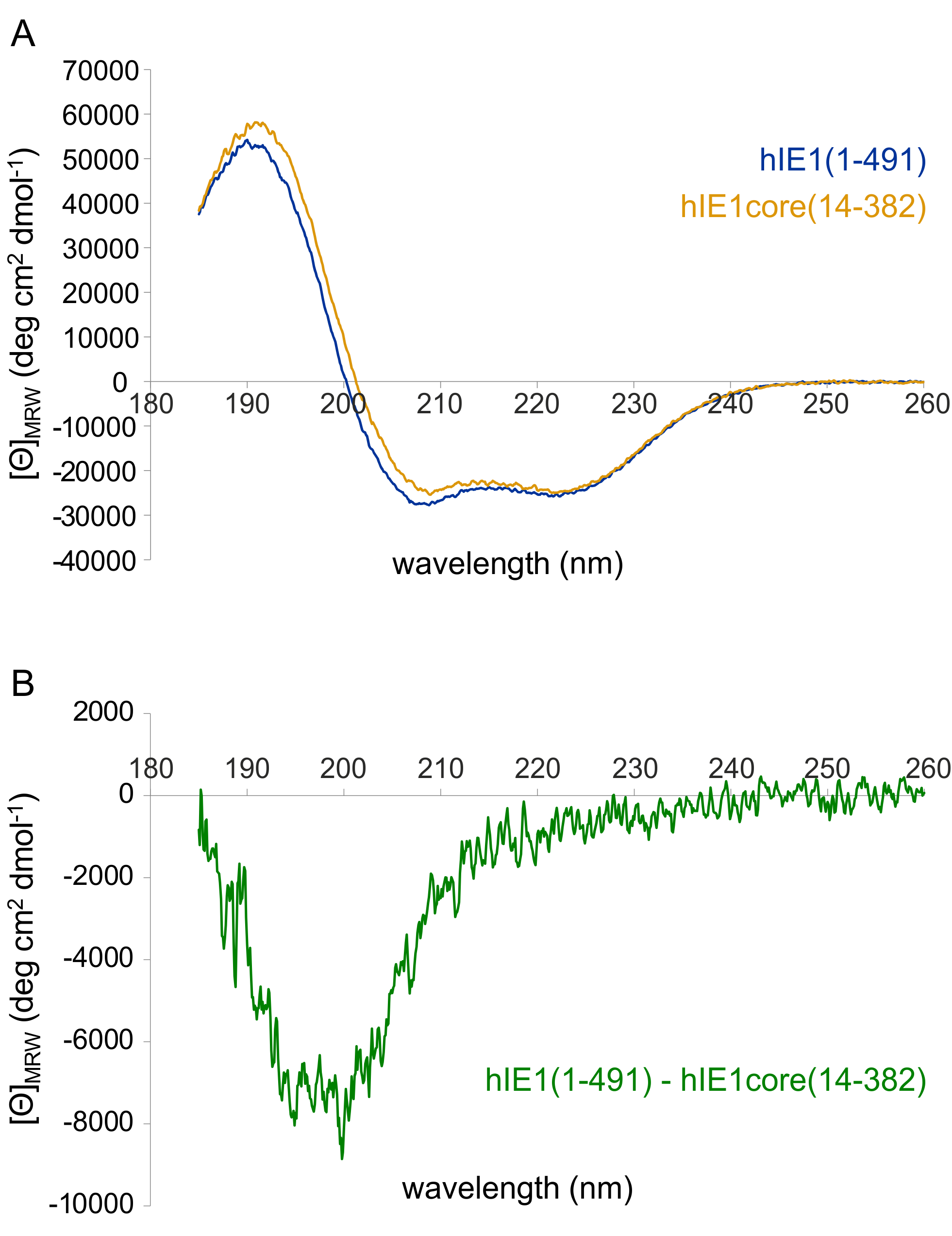

Supplement: Figure S2 — The CD spectra of full-length hIE1(1–491) and hIE1(14–382) are shown in blue and gold, respectively (A). The difference spectrum obtained by subtracting the data of hIE1(14–382) from hIE1(1–491) is shown in green (B). The difference spectrum exhibits strong random coil characteristics with its minimum at a wavelength around 200 nm and therefore confirms the intrinsically disordered nature of the terminal regions. (TIF) [file ppat.1004512.s002.tif]

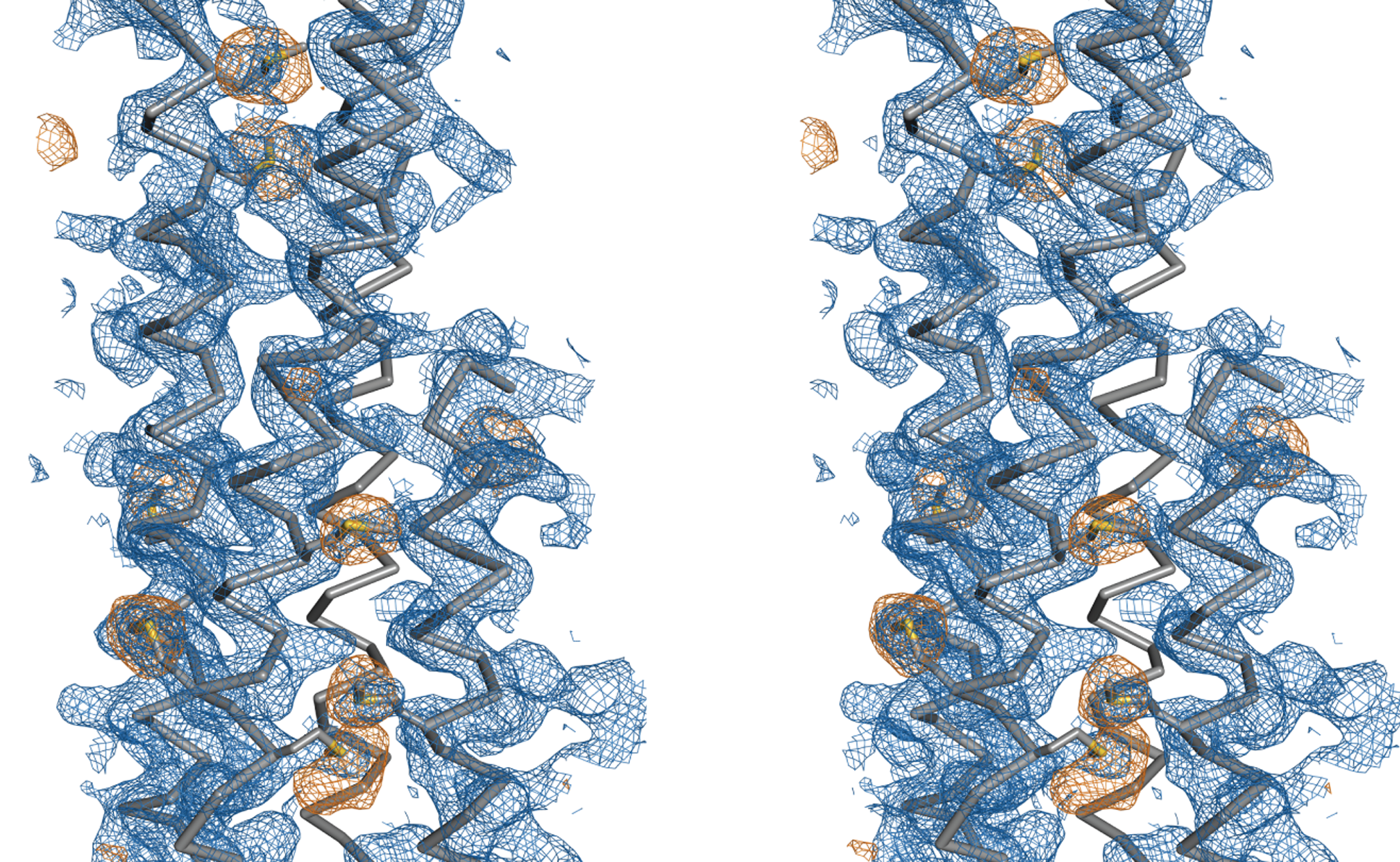

Supplement: Figure S3 — Stereo image of a representative section of the experimentally phased electron density map used for model building. Protein phases in space group P21 were derived from a 3.5 Å MAD dataset collected from a gold-soaked crystal and combined with 3.1 Å data from a SeMet SAD dataset collected at the peak wave-length of Se (Table S1). Extensive four-fold non-crystallographic symmetry averaging was applied. The protein density (contoured at 1σ) is shown in blue and the anomalous density calculated from the anomalous difference signal in the SeMet peak dataset (contoured at 3σ) in orange. The helical structure of the protein is clearly visible and the positions of the Se-atoms coincide with the locations of the peaks in the anomalous density map. (TIF) [file ppat.1004512.s003.tif]

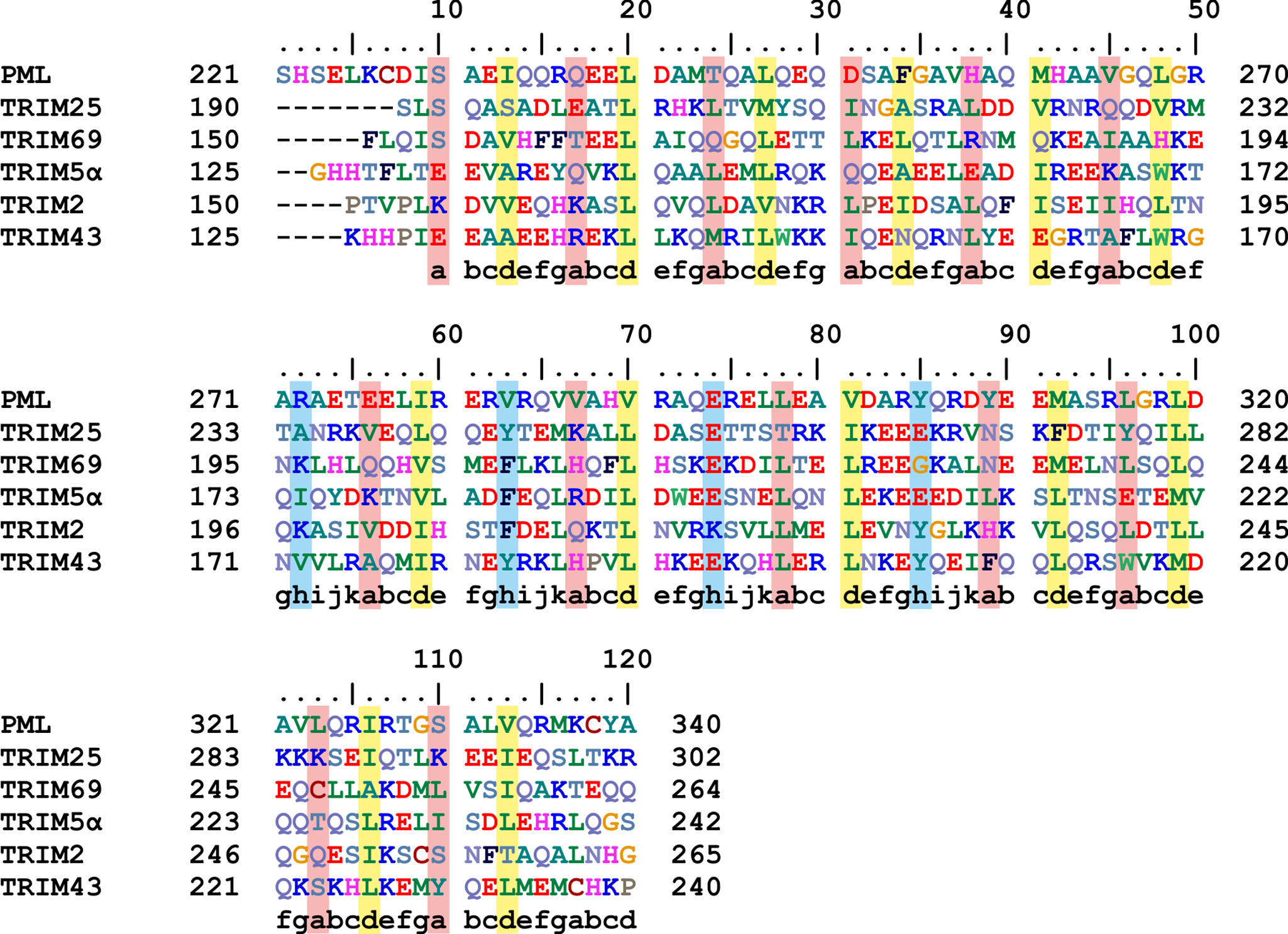

Supplement: Figure S4 — Multiple sequence alignment of the coiled-coil region of different TRIM proteins. The a and d position of the heptad and hendecad repeats and the h position present only in the hendecad repeats are highlighted. (TIF) [file ppat.1004512.s004.tif]

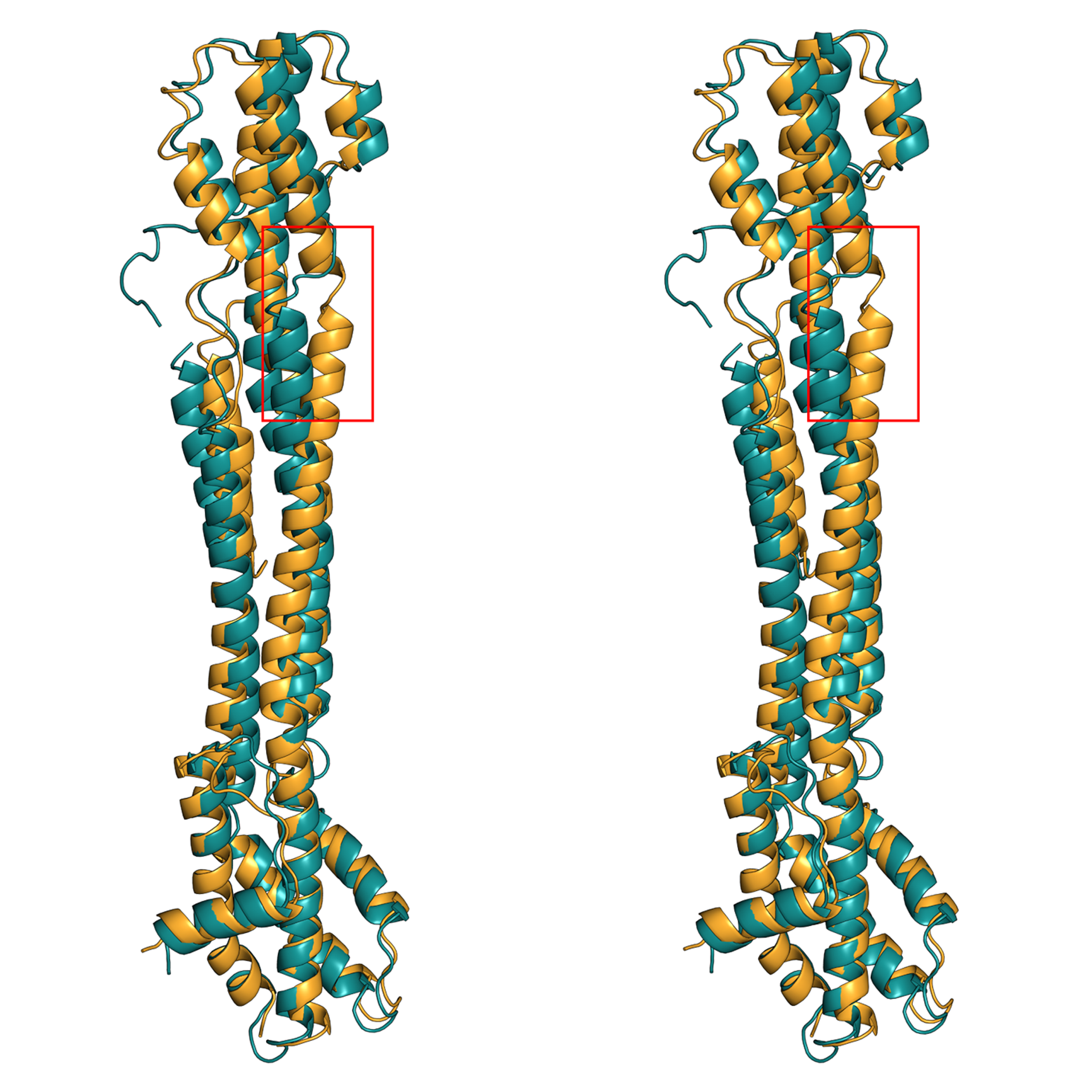

Supplement: Figure S5 — Stereorepresentation showing the superposition of the two monomers that are present in dimeric rhIE1CORE in the final refined structure in space group P43. When considering the Cα positions from the α-helical segments, the two monomers can be superimposed with an r.m.s. deviation of 2.13 Å. The area with the highest deviation, namely, the loop between helices H8 and H9, is marked by a red box (see also Figure S6). The molecules were superposed with the program LSQKAB [66]. (TIF) [file ppat.1004512.s005.tif]

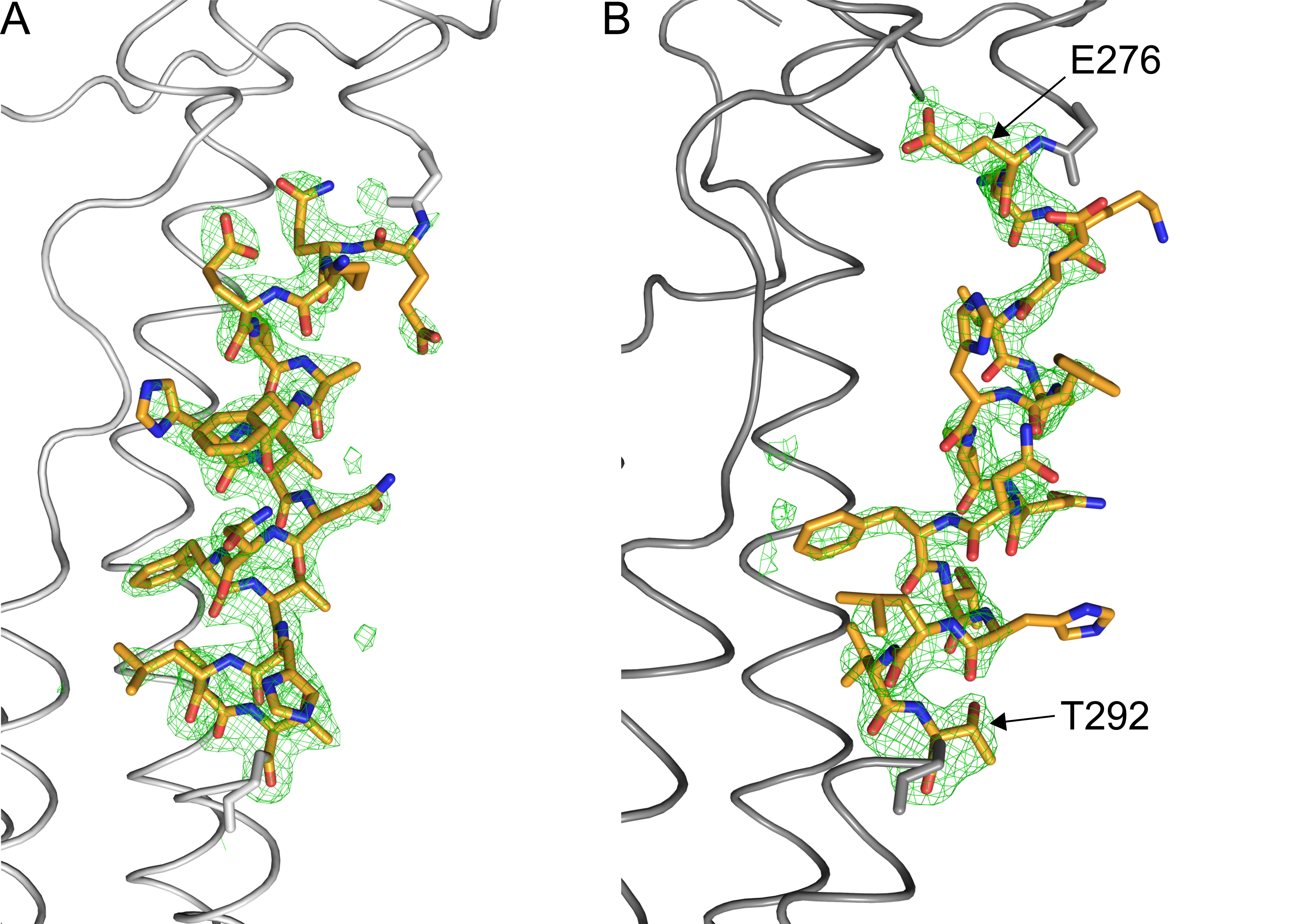

Supplement: Figure S6 — Simulated annealed OMIT difference density map illustrating a difference in the kink of the loop formed between helices H8 and H9 in the two monomers (panel A and B) of dimeric rhIE1CORE (see also Figure S5). The maps were generated with program PHENIX and residues Glu276 to Thr292 omitted during refinement and subsequent difference density calculation. The σA-weighted mFo-DFc electron density is displayed at a 3σ level. (TIF) [file ppat.1004512.s006.tif]

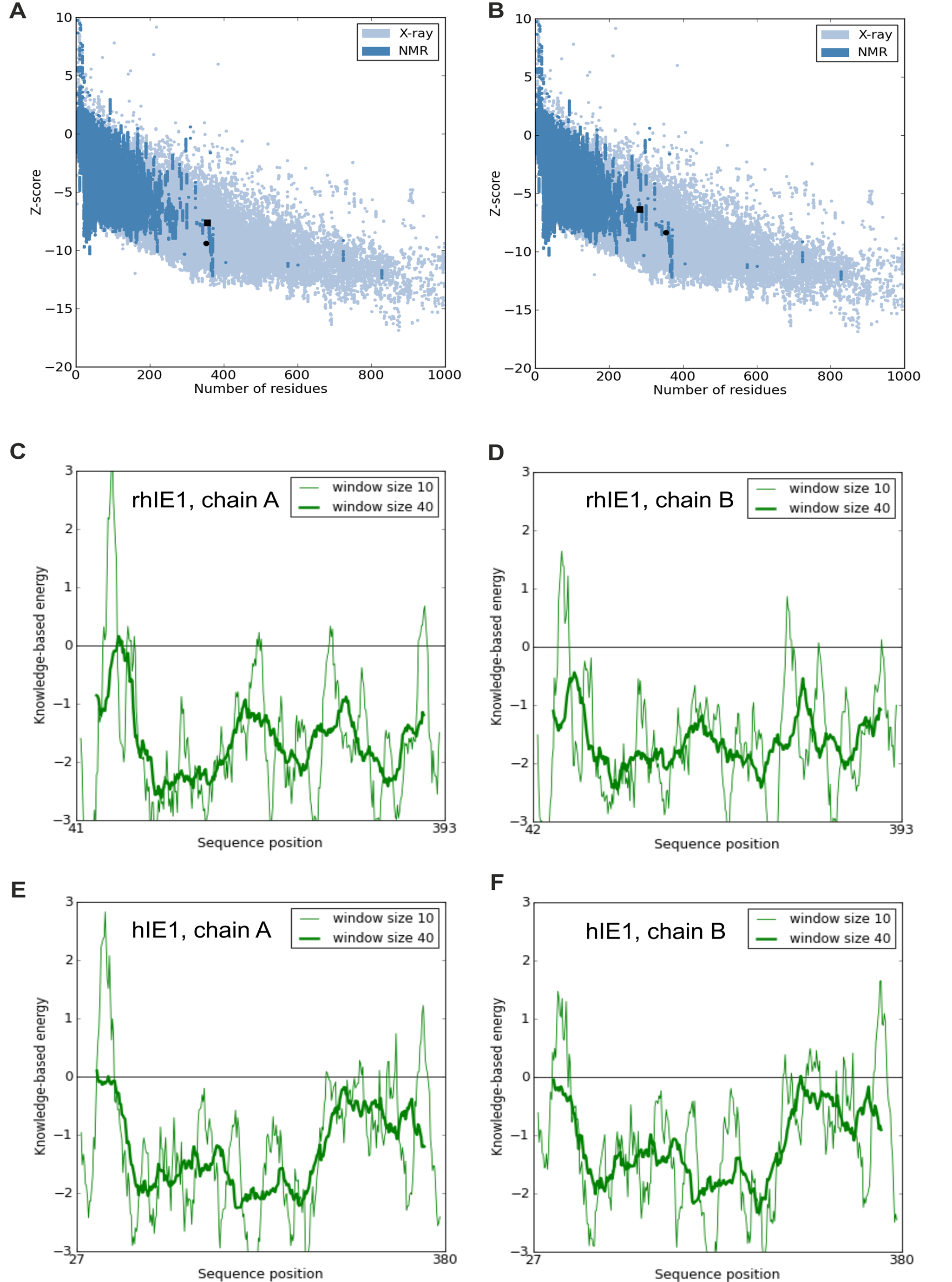

Supplement: Figure S7 — Structure validation of the hIE1 model and comparison of the structural parameters to the rhIE1 template crystal structure. (A, B) Overall structure quality as indicated by the Z-score. The Z-scores of rhIE1 (A) and hIE1 (B) are displayed in a plot that contains the Z-scores of all experimentally determined protein chains in the PDB. In this plot, groups of structures from different sources (X-ray, NMR) are distinguished by different colors. Z-scores for IE1 chain A and B are denoted by a dot and a rectangle symbol, respectively. Note that the Z-scores of the crystal structure and model are very similar and well within the range expected for protein structures of this size. (C)–(F) Local structure quality evidenced by a knowledge-based energy as a function of amino acid sequence position. The plots are smoothed by calculating the average energy over each 10- residue fragment (thin line) or 40-residue fragment (thick line) along the peptide chain. The latter plots are similar for rhIE1 (C, D) and hIE1 (E, F) and exhibit favorable negative energy values along the entire peptide chains. This indicates that the modeling procedure has not placed residues in an unfavorable environment. (TIF) [file ppat.1004512.s007.tif]
