# Supplementary material for: Crystal Structure of Cytomegalovirus IE1 Protein Reveals Targeting of TRIM Family Member PML via Coiled-Coil Interactions
Source: PLoS Pathog. 2014 Nov 20;10(11):e1004512. doi: 10.1371/journal.ppat.1004512 (PMC4239116; doi:10.1371/journal.ppat.1004512)
Supplement: Table S1 — Data collection, phasing and refinement statistics. (DOC) [file ppat.1004512.s008.doc]

**Table S1 - Data collection, phasing and refinement statistics**

|  | Native Dehydrated^a^ |  | Gold^a^ |  | SeMet^a,b^ | Native Initial^a^ |
| --- | --- | --- | --- | --- | --- | --- |
| **Data collection** |  |  |  |  |  |  |
| Space group | P4_3_ |  | P2_1_ |  | P2_1_ | P2_1_ |
| Cell dimensions   |  |  |  |  |  |  |
| *a*, *b*, *c* (Å) | 56.5,  56.5,  276.9 |  | 58.0,  278.6,  61.6 |  | 58.0,  278.8,  61.7 | 58.0,  278.6,  61.6 |
|     α, β, γ  (°) | 90, 90, 90 |  | 90, 91.0, 90 |  | 90, 90.9, 90 | 90, 90.9, 90 |
|  |  | *Peak* | *Inflection* | *Remote* | *Peak* |  |
| Wavelength | 0.9184 | 1.0372 | 1.0402 | 1.0346 | 0.9797 | 0.9184 |
| Resolution (Å) | 49-2.3 (2.37‑2.3) ^c^ | 20.0-3.5 (3.58-3.5) | 20.0-3.5 (3.58-3.5) | 20.0-3.5 (3.58-3.5) | 20-3.1 (3.18-3.1) | 20-2.85 (2.92-2.85) |
| *R*_merge_ (%) | 6.0 (59.1) | 8.1 (53.5) | 9.2 (67.1) | 9.2 (73.0) | 10.5 (95.5) | 7.2 (96.5) |
| *I* / σ*I* | 16.9 (1.6) | 11.5 (2.4) | 10.3 (1.8) | 10.2 (1.7) | 9.7 (1.8) | 16.9 (1.7) |
| Completeness (%) | 99.3 (93.7) | 97.6 (92.5) | 97.2 (87.9) | 99.4 (97.9) | 99.1 (99.8) | 99.5 (99.7) |
| Redundancy | 5.9 | 1.9 | 1.9 | 1.9 | 3.3 | 3.7 |
|  |  |  |  |  |  |  |
| **Refinement** |  |  |  |  |  |  |
| Resolution (Å) | 49-2.3 |  |  |  |  |  |
| No. reflections | 37,710 |  |  |  |  |  |
| *R*_work_ / *R*_free_^d^ | 19.73 / 24.96 |  |  |  |  |  |
| No. atoms | 5833 |  |  |  |  |  |
| Protein | 5694 |  |  |  |  |  |
| Water  Ligands | 123  16 |  |  |  |  |  |
| *B*-factors |  |  |  |  |  |  |
| Protein | 61.8 |  |  |  |  |  |
| Water  Ligands | 51.8  89.5 |  |  |  |  |  |
| R.m.s deviations |  |  |  |  |  |  |
| Bond lengths (Å) | 0.008 |  |  |  |  |  |
| Bond angles (°) | 1.00 |  |  |  |  |  |

^a^One crystal was used for data collection and refinement.

^b^SeMet: selenomethionine.

^c^Values in parentheses are for highest-resolution shell.

^d^Rfree was calculated with 5% of the reflection data

Refinement statistics were calculated with the table_one utility of PHENIX.

Ramachandran favored: 98 %

Ramachandran outliers: 0 %
