# Supplementary material for: Crystal Structure of Cytomegalovirus IE1 Protein Reveals Targeting of TRIM Family Member PML via Coiled-Coil Interactions
Source: PLoS Pathog. 2014 Nov 20;10(11):e1004512. doi: 10.1371/journal.ppat.1004512 (PMC4239116; doi:10.1371/journal.ppat.1004512)
Supplement: Table S2 — List of structurally similar proteins of IE1 sorted by their Z-score in descending order. For each hit the PDB code, the RMSD of the overlay, and the number of structurally equivalent residues (Nalgn) is given. Generally, the number of equivalent residues is rather low covering less than 50% of the total length of IE1. This demonstrates that the overall fold in IE1 is rather unique and there are only local structural similarities to other helical proteins. These local hits were further inspected based on their CATH-classification. Most of the hits from the present search are classified as α-helical orthogonal bundle (code 1.10.) or up-down bundle (code 1.20.). Comparison of the third number of the CATH-code shows that these proteins belong to at least 18 different folds indicating that IE1 cannot readily be assigned to any known topology. This is further corroborated by the calculation of a normalized rmsd (normRMSD): For all hits listed in Table S2 the normRMSD values are significantly larger than the threshold of 5 Å [77], which indicates that a protein is significantly structurally different from those proteins previously deposited in the PDB. (DOC) [file ppat.1004512.s009.doc]

**Table S2 - List of structurally similar proteins of IE1 sorted by their Z-score in descending order**

| # | Z-score | PDB | Chain | RMSD | normRMSD | N_aln_ | CATH Classification | Comment |
| --- | --- | --- | --- | --- | --- | --- | --- | --- |
| 1 | 2.154 | 3rco | B | 3.779 | 35.952 | 37 | unassigned | OST-HTH/LOTUS domain in TDRD7 |
| 2 | 1.975 | 1x90 | B | 2.659 | 12.648 | 74 | 1.20.140.40 | Invertase/pectin methylesterase inhibitor family protein |
| 3 | 1.916 | 4hg4 | b | 3.636 | 15.237 | 84 | 3.90.209.20 | Hemagglutinin -like domain |
| 4 | 1.785 | 2fq3 | A | 3.385 | 24.317 | 49 | 1.10.10.10 | "winged helix" repressor DNA binding domain |
| 5 | 1.695 | 2euc | B | 4.904 | 31.386 | 55 | unassigned | Hypothetical protein yfmB |
| 6 | 1.685 | 4hwf | B | 4.293 | 26.054 | 58 | 1.20.58.120 | BAG family molecular chaperone regulator 5 -like domain |
| 7 | 1.645 | 3k4t | C | 3.734 | 27.965 | 47 | unassigned | virion-associated protein P3 from caulimovirus |
| 8 | 1.423 | 2fh0 | A | 3.450 | 21.305 | 57 | 1.10.8.140 | DNA Binding Protein, Hypothetical Protein Mth1615 |
| 9 | 1.303 | 3gwl | A | 2.906 | 26.919 | 38 | 1.20.120.310 | FAD-linked sulfhydryl oxidase -like domain |
| 10 | 1.263 | 2i15 | A | 3.927 | 30.050 | 46 | 1.20.120.510 | uncharacterised family 2 -like domain |
| 11 | 1.204 | 1mn8 | A | 5.009 | 30.933 | 57 | 1.10.150.180 | Retroviral matrix proteins |
| 12 | 0.979 | 3a5t | A | 3.463 | 18.753 | 65 | 1.10.880.10 | Transcription Factor Skn-1; Chain P |
| 13 | 0.941 | 3ora | T | 3.722 | 31.955 | 41 | 1.20.58.110 | 30S ribosomal protein S20 -like domain |
| 14 | 0.912 | 3vh6 | T | 3.553 | 27.792 | 45 | 1.10.20.10 | Histone H2B -like domain |
| 15 | 0.893 | 2j6y | C | 2.065 | 27.957 | 26 | 1.10.1240.30 | Circadian clock protein kaiA -like domain |
| 16 | 0.893 | 2cuj | A | 4.508 | 29.385 | 54 | 1.10.10.10 | "winged helix" repressor DNA binding domain |
| 17 | 0.819 | 4i3m | A | 4.269 | 21.778 | 69 | unassigned | HAMP Domain Conformers |
| 18 | 0.773 | 3vhb | B | 4.487 | 24.299 | 65 | 1.10.490.10 | Hemoglobin -like domain |
| 19 | 0.773 | 2jps | A | 5.739 | 48.098 | 42 | unassigned | Mlp1-binding domain |
| 20 | 0.746 | 1l6l | 7 | 5.728 | 36.005 | 56 | unassigned | Apolipoprotein A-II |
| 21 | 0.737 | 1zrt | D | 5.334 | 22.089 | 85 | 1.10.760.10 | Cytochrome c -like domain |
| 22 | 0.718 | 3edu | A | 4.169 | 10.870 | 135 | 1.20.58.60 | Spectrin protein 1 -like domain |
| 23 | 0.688 | 3fb2 | B | 4.361 | 10.443 | 147 | 1.20.58.60 | Spectrin protein 1 -like domain |
| 24 | 0.640 | 4iej | A | 4.832 | 42.522 | 40 | unassigned | DNA methyltransferase 1 associated protein 1 |
| 25 | 0.590 | 1u5p | A | 3.701 | 8.802 | 148 | 1.20.58.60 | Spectrin protein 1 -like domain |
| 26 | 0.559 | 3pdy | B | 3.626 | 9.117 | 140 | 1.20.58.60 | Spectrin protein 1 -like domain |
| 27 | 0.547 | 3tvh | 1 | 3.603 | 21.138 | 60 | 1.10.1900.20 | 50S ribosomal protein L20 -like domain |
| 28 | 0.483 | 3u0c | B | 6.018 | 16.812 | 126 | unassigned | Type III Secretion First Translocator IpaB |
| 29 | 0.430 | 2wcd | V | 7.037 | 13.838 | 179 | 1.20.1170.10 | UPF0478 protein SH1183 -like domain |
| 30 | 0.408 | 2fj7 | H | 5.354 | 31.410 | 60 | 1.10.20.10 | Histone H2B -like domain |
| 31 | 0.408 | 2gf4 | A | 3.592 | 22.989 | 55 | 1.20.1270.110 | uncharacterised family 40 -like domain |
| 32 | 0.386 | 3r7c | A | 4.439 | 39.063 | 40 | 1.20.120.310 | FAD-linked sulfhydryl oxidase -like domain |
| 33 | 0.379 | 4fm3 | D | 4.986 | 22.793 | 77 | unassigned | hypothetical protein (PA2901) |
| 34 | 0.373 | 2o37 | A | 3.679 | 27.553 | 47 | 1.10.287.110 | DNAJ protein -like domain |
| 35 | 0.372 | 3w4y | C | 4.267 | 41.722 | 36 | 1.20.120.310 | FAD-linked sulfhydryl oxidase -like domain |
| 36 | 0.365 | 2key | A | 4.485 | 29.787 | 53 | 1.10.150.130 | Tyrosine recombinase xerC -like domain |
| 37 | 0.338 | 2d4c | C | 6.001 | 16.633 | 127 | 1.20.1270.60 | MGC162872 protein -like domain 1/2 |
| 38 | 0.318 | 3mbg | C | 4.299 | 44.507 | 34 | 1.20.120.310 | FAD-linked sulfhydryl oxidase -like domain |
| 39 | 0.305 | 2jxj | A | 5.153 | 33.590 | 54 | 1.10.2010.10 | Crustacean hyperglycemic hormone -like domain |
| 40 | 0.233 | 1j0t | A | 5.346 | 40.038 | 47 | 1.10.2010.10 | Crustacean hyperglycemic hormone -like domain |
| 41 | 0.222 | 3hkc | E | 6.350 | 42.985 | 52 | unassigned | RB3 stathmin-like domain |
| 42 | 0.221 | 1q6b | A | 5.252 | 23.701 | 78 | 1.10.1240.30 | Circadian clock protein kaiA -like domain |
| 43 | 0.171 | 2o3l | A | 5.890 | 40.653 | 51 | 1.20.58.300 | Putative uncharacterized protein -like domain |
| 44 | 0.144 | 3opc | B | 4.602 | 18.620 | 87 | 1.20.58.300 | Putative uncharacterized protein -like domain |
| 45 | 0.112 | 1fad | A | 5.735 | 50.468 | 40 | 1.10.533.10 | Unc5a protein -like domain 1/2 |
| 46 | 0.097 | 2z0v | A | 6.387 | 27.756 | 81 | 1.20.1270.60 | MGC162872 protein -like domain 1/2 |
| 47 | 0.028 | 3g9g | A | 7.871 | 17.316 | 160 | unassigned | EFC/F-BAR domain of Syp1 |
| 48 | 0.020 | 4fzs | B | 4.839 | 19.139 | 89 | unassigned | SNX1 BAR domain |
| 49 | 0.012 | 2vho | N | 7.087 | 59.396 | 42 | 1.20.1270.60 | MGC162872 protein -like domain 1/2 |
| 50 | 0.011 | 3qwe | A | 6.760 | 21.437 | 111 | unassigned | GEM interacting protein |

For each hit the PDB code, the RMSD of the overlay, and the number of structurally equivalent residues (N_algn_) is given. Generally, the number of equivalent residues is rather low covering less than 50% of the total length of IE1. This demonstrates that the overall fold in IE1 is rather unique and there are only local structural similarities to other helical proteins. These local hits were further inspected based on their CATH-classification. Most of the hits from the present search are classified as α-helical orthogonal bundle (code 1.10.) or up-down bundle (code 1.20.). Comparison of the third number of the CATH-code shows that these proteins belong to at least 18 different folds indicating that IE1 cannot readily be assigned to any known topology. This is further corroborated by the calculation of a normalized rmsd (normRMSD): For all hits listed in Table S2 the normRMSD values are significantly larger than the threshold of 5 Å [77], which indicates that a protein is significantly structurally different from those proteins previously deposited in the PDB.
