# Supplementary material for: Crystal Structure of Cytomegalovirus IE1 Protein Reveals Targeting of TRIM Family Member PML via Coiled-Coil Interactions
Source: PLoS Pathog. 2014 Nov 20;10(11):e1004512. doi: 10.1371/journal.ppat.1004512 (PMC4239116; doi:10.1371/journal.ppat.1004512)
Supplement: Table S3 — List of oligonucleotides. (DOC) [file ppat.1004512.s010.doc]

**Table S3: Oligonucleotides**

Oligonucleotides for cloning of IE1 variants into prokaryotic expression plasmid pGEX-6P-1

| 5'hIE1co_BamH1 | CATAGGATCCATGGAGAGTTCGGCAAAACGT |
| --- | --- |
| 3'hIE1co_Xho1 | CATACTCGAGTCACTGATCAGCTTTGGAGCG |
| 5'hIE1co_aa14_BamHI | CATAGGATCCCCTGATGAAGGACCGTCTAGC |
| 5'hIE1co_aa20_BamHI | CATAGGATCCAGCAAAGTTCCTCGCCCAGAA |
| 5'hIE1co_aa29_BamHI | CATAGGATCCATGGTTACTAAAGCGACCACATTC |
| 3'hIE1co_aa377_XhoI | CATACTCGAGTCACTCTTCAGCAATTGCGCGCAG |
| 3'hIE1co_aa379_XhoI | CATACTCGAGTCAGTCGCTCTCTTCAGCAATTGC |
| 3'hIE1co_aa382_XhoI | CATACTCGAGTCATTCCTCCTCGTCGCTCTCTTC |
| 3'hIE1co_aa383_XhoI | CATACTCGAGTCAGGCTTCCTCCTCGTCGCTCTC |
| 5'cIE1co_aa15_BamHI | CATAGGATCCCCGGATGAAGGTCCGAGCAGC |
| 5'cIE1co_aa21_BamHI | CATAGGATCCAGCAAAATTCCGCGTCCGGAA |
| 3'cIE1co_aa383_XhoI | CATACTCGAGTTATTCATCTTCATCACTTTCTTC |
| 5'rhIE1co_aa36_BamHI | CATAGGATCCAAACAGGCACGTAAAGATATG |
| 3'rhIE1co_aa395_XhoI | CATACTCGAGTTAATCTTCATTGATTTTATCATT |

Oligonucleotides for cloning of hIE1 and PML variants into eukaryotic expression plasmids pHM971 and pHM1580

| 5’hIE1_BamHI | CATAGGATCCATGGAGTCCTCTGCCAAGAG |
| --- | --- |
| 5’hIE1aa14_BamHI | CATAGGATCCCCTGACGAGGGCCCTTCCTCC |
| 5’hIE1aa20_BamHI | CATAGGATCCTCCAAGGTGCCACGGCCCGAG |
| 3’hIE1_XhoI | CATACTCGAGTTACTGGTCAGCCTTGCTTC |
| 3’hIE1aa377_XhoI | CATACTCGAGTTACTCCTCGGCGATGGCCCGTAG |
| 3’hIE1aa382_XhoI | CATACTCGAGTTACTCTTCCTCATCTGACTCCTC |
| 5'PMLaa103_BamHI | CATAGGATCCGCCCTGGATAACGTCTTTTTCG |
| 3'PMLVI_XhoI | CATACTCGAGTCACCACAACGCGTTCCTCT |

Oligonucleotides for cloning of PML variants into eukaryotic expression plasmid pHM972

| 5'PML_EcoRI | CATAGAATTCTATGGAGCCTGCACCCGCCCG |
| --- | --- |
| 5'PMLaa228_EcoRI | CATAGAATTCTGACATCAGCGCAGAGATCCA |
| 3'PMLaa399_NotI | CATAGCGGCCGCTCATGGATACAGCTGCATCTTTC |
| 3'PMLaa255_NotI | CATAGCGGCCGCTCAGCCAAAGGCACTATCCTGCT |

Oligonucleotides for cloning of hIE1 variants and PML into yeast expression plasmids pGBT9 and pGAD424

| 5'hIE1_EcoRI | CATAGAATTCATGGAGTCCTCTGCCAAGAG |
| --- | --- |
| 5'hIE1_aa14_EcoRI | CATAGAATTCCCTGACGAGGGCCCTTCCTCC |
| 3'hIE1_Sal | TCACGTCGACTTACTGGTCAGCCTTGCTTCTAG |
| 3'hIE1_aa382_SalI | CATAGTCGACTTACTCTTCCTCATCTGACTCCTC |
| 5'PML_EcoRI | CATAGAATTCATGGAGCCTGCACCCGCCCGA |
| 3'PML_SalI  3'PML_aa386_SalI | CATAGTCGACTCACCACAACGCGTTCCTCTC  CATAGTCGACGAGGTCCTGCAGGCGCACCTT |
| 3'PML_aa267_SalI | CATAGTCGACTCACTGGCCGACGGCCGCGTGCAT |
| 3'PML_aa241_SalI | CATAGTCGACGTCCAGCTCCTCCTGTCGCTG |
| 3'PML_aa104_SalI | CATAGTCGACCAGGGCGGGTGTGTCTGCACC |

Oligonucleotides for cloning of hIE1 variants into the pLKO-based lentiviral expression vector

| 5'hIE1_AgeI | CATAACCGGTATGGAGTCCTCTGCCAAGAG |
| --- | --- |
| 5'Myc-hIE1_AgeI | CATAACCGGTATGGAACAAAAACTCATCTCAGAAGAGGATCTGGAGTCCTCTGCCAAGAGAAA |
| 3'hIE1aa382_EcoRI | CATAGAATTCTTACTCTTCCTCATCTGACTCCT |

Oligonucleotides for BAC recombination

| 5’BAC_short | TAGGGATAACAGGGTAATCGATTT |
| --- | --- |
| 5’BAC_hIE1_382 | TGACCTACGGGCCATCGCCGAGGAGTCAGATGAGGAAGAGTAAATTGTAGCCTACACTTTGGCTAGGGATAACAGGGTAATCGATTT |
| 3’BAC_hIE1_382 | AGGAGCTGACACCAGCGGTGGCCAAAGTGTAGGCTACAATTTACTCTTCCTCATCTGACTCCTCAACCAATTAACCAATTCTGATTAG |
| 3’BAC_hIE1_382_short | AGGAGCTGACACCAGCGGTG |
| 5’Intron3/pKD13 | AAAGATGTCCTGGCAGAACTCGGTAAGTCTGTTGACATGTATGTGATGTAGTGTAGGCTGGAGCTGCTTC |
| 3’Exon 4/pkd13 | TAGTTTACTGGTCAGCCTTGCTTCTAGTCACCATAGGGTGGGTGCTCTTGATTCCGGGGATCCGTCGACC |

Oligonucleotides for real-time PCR

| 5'gB_forw | CTGCGTGATATGAACGTGAAGG |
| --- | --- |
| 3'gB_rev | ACTGCACGTACGAGCTGTTGG |
| CMV gB FAM/TAMRA | CGCCAGGACGCTGCTACTCACGA |
